# Deletion of the rodent malaria ortholog for falcipain-1 highlights differences between hepatic and blood stage merozoites

**DOI:** 10.1371/journal.ppat.1006586

**Published:** 2017-09-18

**Authors:** Christine S. Hopp, Brandy L. Bennett, Satish Mishra, Christine Lehmann, Kirsten K. Hanson, Jing-wen Lin, Kimberly Rousseau, Filomena A. Carvalho, Wouter A. van der Linden, Nuno C. Santos, Matthew Bogyo, Shahid M. Khan, Volker Heussler, Photini Sinnis

**Affiliations:** 1 Department of Molecular Microbiology & Immunology, Johns Hopkins Bloomberg School of Public Health, Baltimore, Maryland, United States of America; 2 Department of Microbiology, New York University School of Medicine, New York, New York, United States of America; 3 Bernhard Nocht Institute for Tropical Medicine, Hamburg, Germany; 4 Instituto de Medicina Molecular, Faculdade de Medicina Universidade de Lisboa, Lisbon, Portugal; 5 Department of Parasitology, Leiden Malaria Research Group, Leiden University Medical Center, Leiden ZA, The Netherlands; 6 Departments of Pathology and Microbiology and Immunology, Stanford University School of Medicine, Stanford, CA, United States of America; 7 Institute of Cell Biology, University of Bern, Bern, Switzerland; Agency for Science, Technology and Research - Singapore Immunology Network, SINGAPORE

## Abstract

Proteases have been implicated in a variety of developmental processes during the malaria parasite lifecycle. In particular, invasion and egress of the parasite from the infected hepatocyte and erythrocyte, critically depend on protease activity. Although falcipain-1 was the first cysteine protease to be characterized in *P*. *falciparum*, its role in the lifecycle of the parasite has been the subject of some controversy. While an inhibitor of falcipain-1 blocked erythrocyte invasion by merozoites, two independent studies showed that falcipain-1 disruption did not affect growth of blood stage parasites. To shed light on the role of this protease over the entire *Plasmodium* lifecycle, we disrupted berghepain-1, its ortholog in the rodent parasite *P*. *berghei*. We found that this mutant parasite displays a pronounced delay in blood stage infection after inoculation of sporozoites. Experiments designed to pinpoint the defect of *berghepain-1* knockout parasites found that it was not due to alterations in gliding motility, hepatocyte invasion or liver stage development and that injection of *berghepain-1* knockout merosomes replicated the phenotype of delayed blood stage growth after sporozoite inoculation. We identified an additional role for berghepain-1 in preparing blood stage merozoites for infection of erythrocytes and observed that *berghepain-1* knockout parasites exhibit a reticulocyte restriction, suggesting that berghepain-1 activity broadens the erythrocyte repertoire of the parasite. The lack of berghepain-1 expression resulted in a greater reduction in erythrocyte infectivity in hepatocyte-derived merozoites than it did in erythrocyte-derived merozoites. These observations indicate a role for berghepain-1 in processing ligands important for merozoite infectivity and provide evidence supporting the notion that hepatic and erythrocytic merozoites, though structurally similar, are not identical.

## Author summary

Malaria affects hundreds of millions of people and is the cause of hundreds of thousands of deaths each year. Infection begins with the inoculation of sporozoites into the skin during the bite of an infected mosquito. Sporozoites subsequently travel to the liver, where they invade and replicate in hepatocytes, eventually releasing the stage of the parasite that is infectious for red blood cells, termed merozoites. Hepatic merozoites initiate blood stage infection, the stage that is responsible for the clinical symptoms of malaria. The blood stage of the parasite grows through repeated rounds of invasion, development and egress of blood stage merozoites, which then continue the cycle. Proteases are among the enzymes that are essential for parasite survival and their functions range from invasion of red blood cells, to the breakdown of red cell hemoglobin, to the release of parasites from red cells. As the function of the cysteine protease falcipain-1 in the lifecycle of the human malaria parasite *Plasmodium falciparum* remains poorly understood, we decided to study berghepain-1, the orthologue of the rodent malaria parasite *P*. *berghei* by generating a *berghepain-1* deletion parasite. Using this mutant, we demonstrate that berghepain-1 has a critical role in both hepatic and erythrocytic merozoite infectivity. Little is known about differences between these two types of merozoites and our data leads us to conclude that these merozoites are not identical.

## Introduction

Malaria, caused by parasites of the genus *Plasmodium*, continues to be a global health problem, causing significant morbidity and mortality particularly in resource poor settings [1]. Human infection begins with the injection of sporozoites into the skin where, using gliding motility, they find and enter the blood stream, carrying the parasites to the liver [2]. Here, they invade hepatocytes and develop into exo-erythrocytic forms (EEFs), replicating to produce thousands of hepatic stage merozoites. Once mature, these liver stage merozoites bud from the infected hepatocytes and enter the blood stream in packets termed merosomes [3]. Hepatic merozoites are released from merosomes and invade erythrocytes, where they develop and divide to produce daughter blood stage merozoites. Infected red blood cells eventually rupture to release the newly formed merozoites that can then go on to invade new red blood cells. Thus, an iterative cycle of parasite replication is established, leading to high numbers of parasite-infected erythrocytes in the host blood stream and clinical symptoms of malaria. A proportion of erythrocytic stage parasites differentiate to sexual stage parasites, which are transmitted to the mosquito as it takes a blood meal. Fertilization occurs in the mosquito midgut and the parasite migrates across the midgut wall to form oocysts containing sporozoites that are released and invade the salivary glands to be injected during the next blood meal. With the emergence of insecticide-resistant mosquitoes and parasites that are increasingly resistant to available antimalarial drugs, there is an urgent need for the identification of new drug targets.

Cysteine proteases play key roles at multiple stages of the *Plasmodium* life cycle, including functions in host cell invasion [4–6], hemoglobin degradation [7,8] and facilitation of parasite egress from hepatocytes [3] and erythrocytes through cleavage of both parasite proteins [9,10] and erythrocyte ankyrin [11]. Of the 33 putative cysteine proteases encoded in the *P*. *falciparum* genome [12], the falcipain family of papain-like cysteine proteases contains four members, with falcipain-2 and -3 having well-established roles in the degradation of host erythrocyte hemoglobin in the parasite food vacuole [13–17]. While falcipain-1 was the first cysteine protease to be characterized in *P*. *falciparum* [18], its physiological role in the lifecycle of the parasite still remains poorly understood. Compared to falcipain-2 and -3, which are similar in sequence (68% of sequence identity), falcipain-1 shares only 38–40% of sequence identity to the other falcipains. Falcipain-1 was detected in the transcriptome [19] and proteomes of asexual and sexual erythrocytic stages of the parasite, as well as in the sporozoite stage [20–22]. Based on the generation of an inhibitor for falcipain-1, it was suggested that this protease plays an important role in merozoite invasion of erythrocytes [23]. These data are consistent with many lines of evidence showing that proteases are required for host cell invasion by Apicomplexan parasites, specifically for processing of surface proteins to expose adhesive domains and to release adhesive interactions ([24–28], reviewed in [9]) and with a previous study that found deletion of the rodent ortholog of falcipain-1 resulted in a blood stage growth defect [13]. Interpretation of these results has been complicated by two subsequent studies which found that deletion of falcipain-1 in *P*. *falciparum* lines 3D7 and D10 did not impact growth of erythrocytic stages of the parasite [17,29,30].

Given the controversy surrounding the role of falcipain-1 in merozoite invasion, and the possibility that it might function in mosquito stages, we proposed to shed light on the role of falcipain-1 by studying its ortholog in the rodent parasite *P*. *berghei*. Rodent malaria parasites have a smaller repertoire of falcipain-family proteases compared to *P*. *falciparum* with only one ortholog with similarity to falcipain-2/-3 identified. However, orthologs of falcipain-1 exist in all *Plasmodium* species studied to date [13,15]

In the present study, we generated a deletion mutant of *berghepain-1*, (PBANKA_132170) as well as an epitope-tagged berghepain-1 parasite. Our results support a role for berghepain-1 in hepatic and erythrocytic merozoite infection of erythrocytes and in particular point to a function of this protease in infection of mature red blood cells. Importantly, we find that hepatic merozoite infectivity is impaired more than infectivity of blood stage merozoites, indicative of differences between these two types of merozoites.

## Results

### Berghepain-1 is not required in the mosquito vector

To investigate whether berghepain-1 is critical *P*. *berghei* development we generated *berghepain-1* deletion or ‘knockout’ (BP1-KO) parasites by replacing the *berghepain-1* open reading frame with a drug selection cassette, using the flanking untranslated regions to target the locus for homologous recombination (S1 Fig). Two independent transfections were performed and verification of *berghepain-1* deletion was performed by PCR and protein deletion was verified using a probe that binds to the berghepain-1 protein (S2 Fig). One clone from each transfection, designated BP1-KO clone 1 and BP1-KO clone 2, were characterized. Data shown displays combined results obtained using both clones, when indicated. To control for effects due to manipulation of the genetic locus, a berghepain-1 control parasite (BP1-CON) was generated using a targeting plasmid containing the entire *berghepain-1* gene with its endogenous 5' and 3' regulatory elements, as well as the selectable marker cassette (S1 Fig). Each construct was transfected into GFP-expressing *P*. *berghei* ANKA clone 507cl1 [31], a strain lethal to mice.

**Fig 1:**
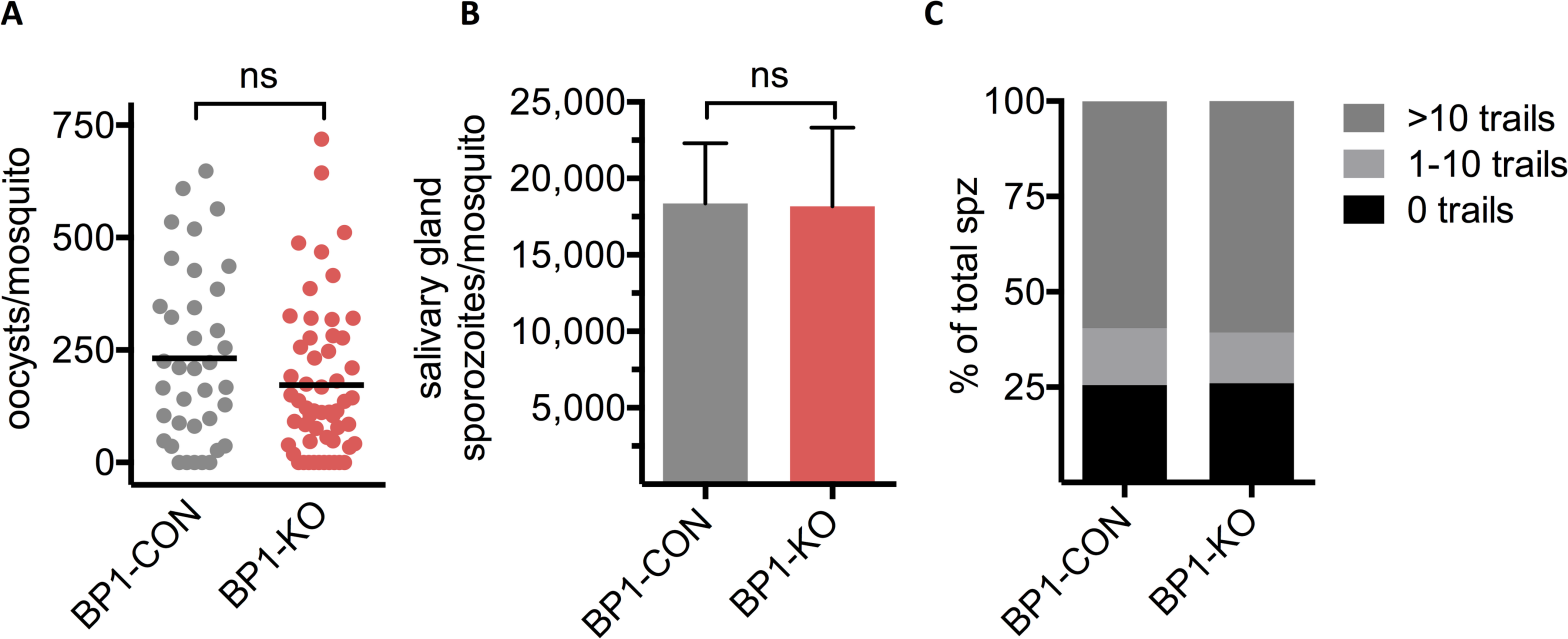
*Berghepain-1* knockout parasites develop normally in the mosquito and sporozoites are motile. **A. Oocyst formation.** Midguts were dissected 13 days after the infectious blood meal and GFP-positive oocysts were counted. Horizontal lines represent mean values. Shown are pooled results from two independent experiments using BP1-CON and BP1-KO clone 1, and one experiment using BP1-CON and BP1-KO clone 2. **B. Salivary gland sporozoite numbers.** Salivary glands were isolated from 20 mosquitoes, 19 days after the infectious blood meal, and sporozoites were counted. Shown are the mean and standard deviation of sporozoite numbers per mosquito, obtained from four independent experiments using BP1-CON and BP1-KO clone 1, and one experiment using BP1-CON and BP1-KO clone 2. **C. Gliding motility**. BP1-CON and BP1-KO sporozoites were allowed to glide for 1 h at 37°C on coated glass coverslips and trails were visualized by detection of shed surface protein CSP. A total number of 2181 BP1-CON and 3189 BP1-KO sporozoites with and without trails were counted and the percent of sporozoites associated with the indicated number of circular trails is shown. Both clones of BP1-KO parasites were analyzed and results were pooled.

*A*. *stephensi* mosquitoes were infected with both *berghepain-1* knockout clones, as well as the control line BP1-CON and development from oocyst to salivary gland sporozoites was followed. Both BP1-KO clone 1 and BP1-KO clone 2 parasites were compared to the control line BP1-CON and were found to generate normal numbers of oocysts (Fig 1A), and salivary gland sporozoites (Fig 1B), indicating that berghepain-1 does not have a crucial role at these stages of the parasite life cycle.

Gliding motility is key for sporozoite migration out of the inoculation site and for infection of hepatocytes upon arrival in the liver [[32,33], reviewed in [2]]. To test whether *berghepain-1* knockout sporozoites are motile and viable, we performed an *in vitro* gliding motility assay, quantifying the proportion of motile salivary gland sporozoites and the trails they leave behind. As sporozoites glide, they deposit trails of surface proteins, such as the circumsporozoite protein (CSP), which can be stained and trails can be manually counted [34]. Motility of BP1-KO sporozoites was comparable to that of control parasites, indicating that berghepain-1 does not have a critical role in sporozoite gliding motility (Fig 1C).

### Delayed prepatent period of *berghepain-1* knockout sporozoites

To investigate the infectivity of *berghepain-1* knockout sporozoites, we inoculated mutant and control sporozoites intravenously (i.v.) into mice and determined the time to detectable blood stage infection, termed the prepatent period, by Giemsa-stained blood smears. Typically in mice, an i.v. inoculum of 100 or 1,000 wild-type sporozoites results in detectable blood stage parasites with a prepatent period of three and four days, respectively, with each day of delay indicating an approximate 10-fold reduction in the infectious inoculum or in the downstream events ultimately leading to detectable blood-stage parasites [35]. Following inoculation of 100 to 10,000 sporozoites into C57BL/6 and Swiss Webster mice, many of the mice inoculated with BP1-KO clone 1 or clone 2 sporozoites failed to develop blood stage parasitemia (Table 1). Of those mice that developed parasitemia, the prepatent period of BP1-KO sporozoites was consistently delayed by approximately four days compared to mice inoculated with the same number of BP1-CON sporozoites (Table 1).

**Table 1:** *In vivo* infectivity of *berghepain-1* knockout sporozoites as determined by prepatent period. C57BL/6 or Swiss Webster mice were inoculated i.v. with the indicated number of sporozoites. Blood smears were examined daily starting on day 3 after sporozoite inoculation and mice were considered negative if parasites were not detected by day 20. For C57BL/6 mice, data from three independent experiments are pooled; two with BP1-KO clone 1 and one with BP1-KO clone 2. For Swiss Webster mice, data from two independent experiments using BP1-KO clone 1 are pooled.

Mouse line	Parasite	Dose	#mice infected #mice inoculated	Prepatent period (days)
C57BL/6	BP1-CON	100	10/10 (100%)	3.8
	BP1-CON	1,000	10/10 (100%)	3.0
	BP1-KO	100	6/15 (40%)	7.5
	BP1-KO	1,000	13/15 (86%)	7.0
Swiss Webster	BP1-CON	1,000	10/10 (100%)	4.2
	BP1-CON	10,000	10/10 (100%)	3.1
	BP1-KO	1,000	3/15 (20%)	7.5
	BP1-KO	10,000	7/10 (70%)	8.5

### *Berghepain-1* knockout sporozoites invade and develop normally in the liver

**Fig 2:**
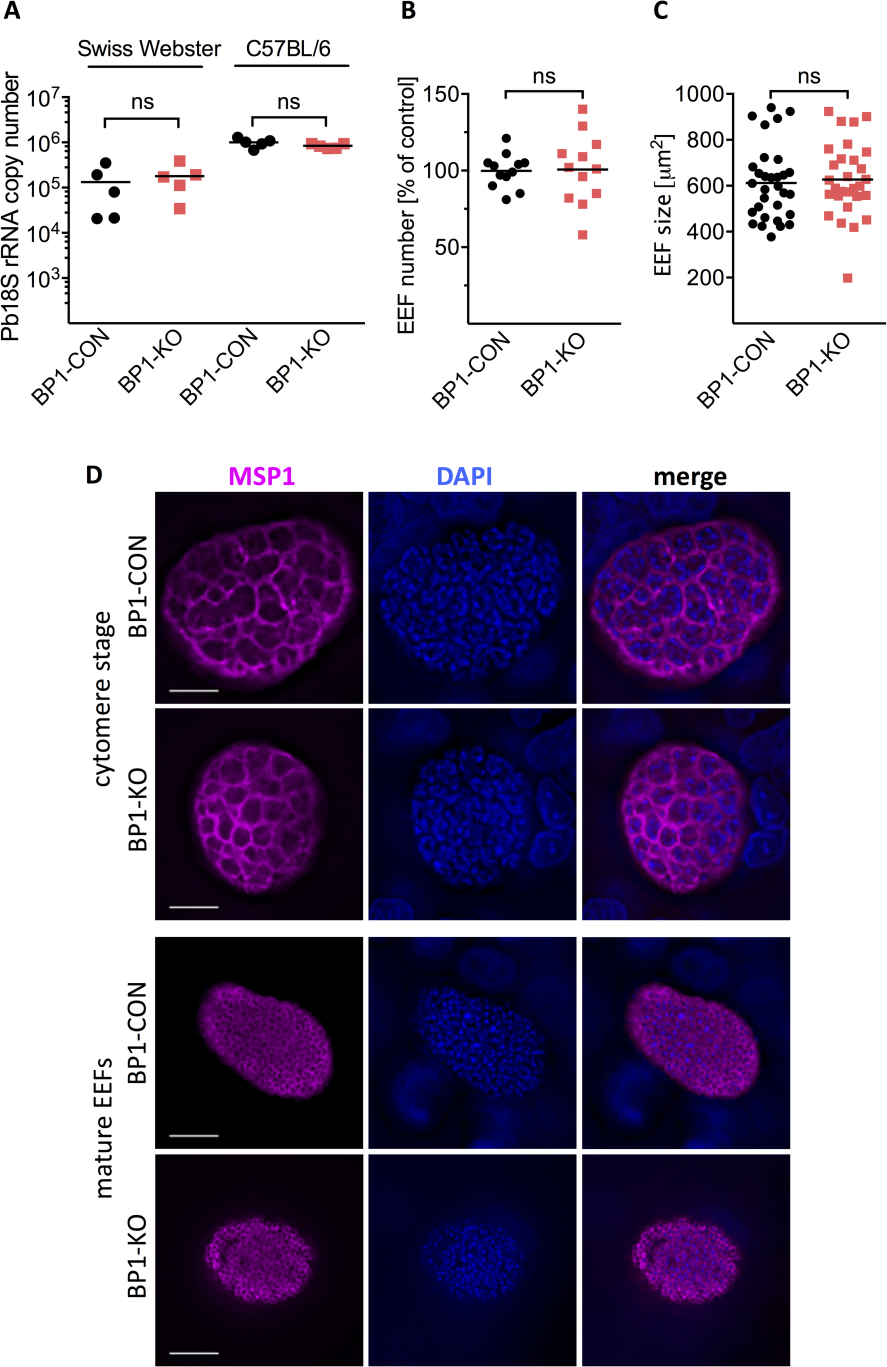
Normal *in vivo* and *in vitro* growth of *berghepain-1* knockout liver stage parasites. **A. Development of *berghepain-1* knockout liver stage parasites *in vivo*.** 10,000 BP1-CON or BP1-KO sporozoites were injected i.v. into Swiss Webster or C57BL/6 mice. RT-qPCR analysis of liver RNA 40 h after infection found no significant reduction of BP1-KO liver stage growth as measured by parasite 18S rRNA copy number. Results from one representative experiment using BP1-CON and BP1-KO clone 1, from a total of three experiments, is shown. **B. & C. Development and size of *berghepain-1* knockout EEFs *in vitro*.** HepG2 cells were infected with BP1-CON and BP1-KO parasites and at 60 h post infection the number of EEFs was counted (**B**) and their sizes measured (**C**). Pooled results of two independent experiments, one using BP1-KO clone 1 and one using BP1-KO clone 2 are shown. **D. Immunofluorescene assays of late liver stage *berghepain-1* knockout parasites.** HepG2 cells infected with either BP1-CON or BP1-KO clone 1 sporozoites were fixed 56 h (cytomere stage) and 72 h (mature EEF) post-infection. EEFs were stained for MSP1 to visualize the parasite membrane (magenta) and DNA was stained with DAPI (blue). Scale bars: 10 μm.

Given the delay in prepatent period after injection of BP1-KO sporozoites compared to controls, we set out to systematically investigate sporozoite infection and development in the liver, to identify the stage at which berghepain-1 is required. To assess the liver infectivity of BP1-CON and BP1-KO parasites *in vivo*, 10,000 BP1-KO or BP1-CON salivary gland sporozoites were injected i.v. or intradermally (i.d.) and 40 h post infection livers were harvested for quantification of *Pb*18S rRNA. No significant reduction of BP1-KO liver stage growth compared to BP1-CON was found by either route of inoculation (Fig 2A and S3 Fig, respectively), suggesting that berghepain-1 is not required for sporozoite exit from the dermis or infection and development in the liver.

To further investigate EEF development, BP1-CON and BP1-KO sporozoites were allowed to invade HepG2 cells *in vitro*. At 60 h post infection, cells were fixed and EEFs were manually counted and their diameter was measured. BP1-KO parasites showed robust development *in vitro* and no differences in the number or size of EEFs were observed (Fig 2B and 2C). Following this, we imaged the different stages of EEF development *in vitro*. After an initial growth phase, nuclear division occurs and the parasite membrane invaginates to form the cytomere stage [36]. Subsequently, individual hepatic merozoites bud from each cytomere to form a mature EEF full of hepatic merozoites. MSP1, the major surface protein of merozoites [37] is observed lining the cytomeres and then localizes to individual hepatic merozoites [38]. At 56 h and 72 h post infection, BP1-CON and BP1-KO EEFs were stained for MSP1, which showed that the cytomere and late schizont stages in BP1-KO parasites are morphologically indistinguishable to control EEFs (Fig 2D). The numbers of cytomere stage and fully mature EEFs were also counted in these experiments and there were no differences between controls and BP1-KO parasites. Overall, these data suggest that berghepain-1 is not required for liver stage growth or maturation of *P*. *berghei*.

### Normal merosome formation and parasitophorous vacuole rupture in *berghepain-1* knockout liver stage parasites

**Fig 3:**
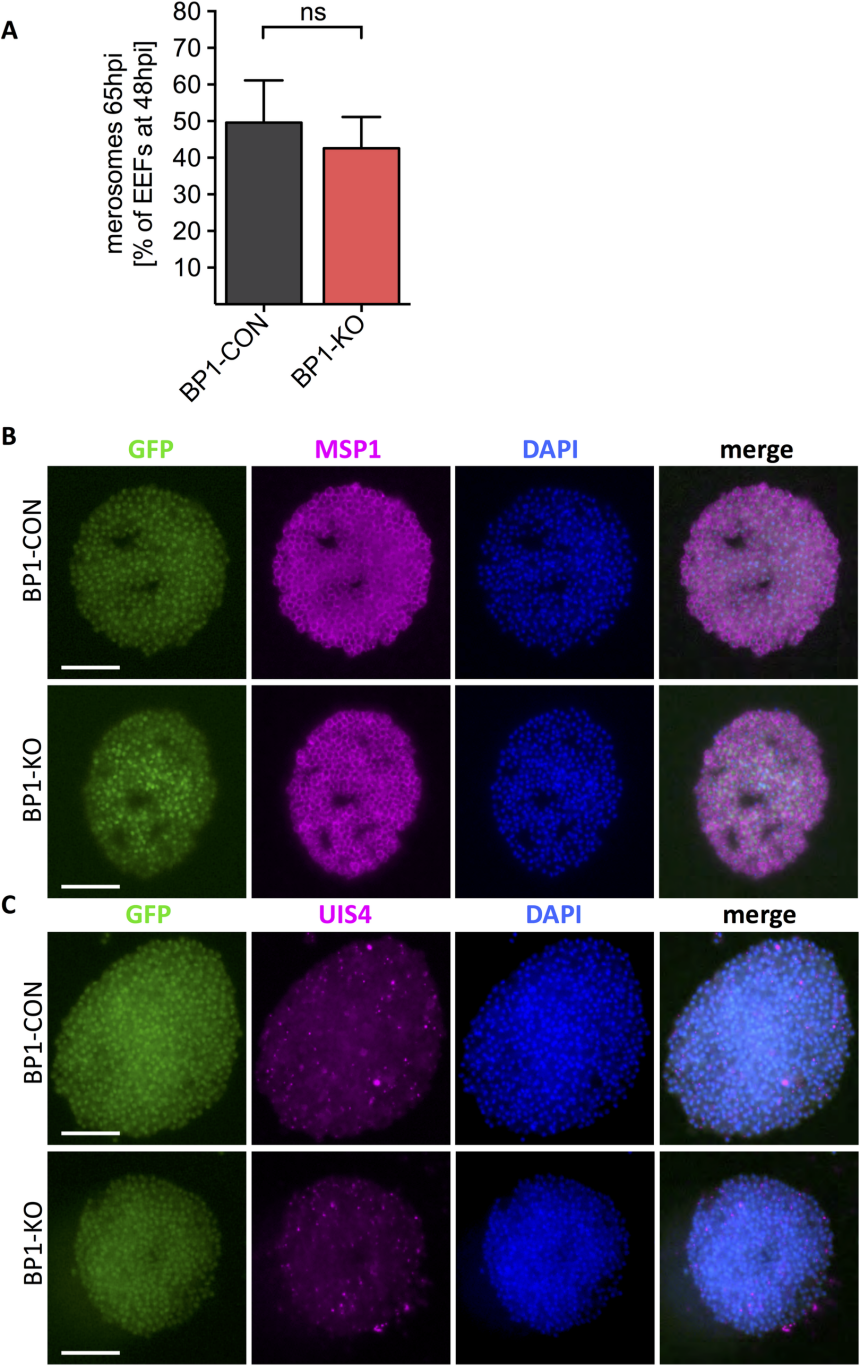
Normal merosome formation in *berghepain-1* knockout liver stage parasites. HepG2 cell monolayers were infected with BP1-CON and BP1-KO parasites, and merosomes were quantified and evaluated by immunofluorescence assays. **A. Quantification of merosomes.** The number of merosomes from BP1-CON and BP1-KO parasites released into the culture supernatant at 65 h post infection were counted and expressed as percentage of EEFs present at 48 h post infection. Results of four experiments performed using BP1-CON and BP1-KO clone 2 are pooled and shown. **B-C**. **Immunofluorescene assays**. Merosomes of BP1-CON and BP1-KO clone 1 parasites were collected at 65 h post infection and stained for MSP1, to visualize individual merozoites, or UIS4 (up-regulated in infective sporozoites), to visualize the parasitophorous membrane (magenta). Parasites express GFP (green) and DNA was stained with DAPI (blue). Scale bars: 15 μm.

In order to successfully release infective merozoites into the blood, the parasitophorous vacuole membrane (PVM) enclosing the EEF ruptures to release hepatic merozoites into the cytoplasm of the hepatocyte. These then bud from the hepatocyte in packets termed merosomes to enter the blood stream [3,39]. Given the involvement of cysteine proteases in both PVM rupture and the release of merosomes [3], we speculated that berghepain-1 might be involved in this process. We quantified the number of merosomes and detached cells, which contain ruptured EEFs, released into culture supernatants at 65 h post infection and normalized this number to the number of EEFs present at 48 h post infection. For clarity, we will refer to merosomes and detached cells as merosomes throughout the manuscript. After 65 h of in vitro culture, we found that compared to BP1-CON parasites, similar numbers of merosomes were produced by BP1-KO clone 2 (Fig 3A) and BP1-KO clone 1 (S4 Fig).

To evaluate merosome morphology and loss of the PVM in *berghepain-1* knockout merosomes we fixed and stained merosomes with antibodies to MSP1, to visualize individual merozoites, and UIS4 [up-regulated in infective sporozoites gene 4 [40]], a marker for the PVM [41]. A previous study demonstrated that PV rupture occurs in the infected hepatocyte and is immediately followed by merosome formation [39] so we would not expect to see UIS4 staining on properly developed merosomes. Staining for MSP-1 showed normal segregation of merozoite membranes (Fig 3B) and staining for UIS4, showed normal loss of the parasitophorous membrane (Fig 3C), suggesting that *berghepain-1* knockout merosomes have normal morphology. Together, these data demonstrate that BP1-KO parasites develop into EEFs and form morphologically normal merozoites and merosomes.

### Berghepain-1 is critical for hepatic merozoite infectivity

**Fig 4:**
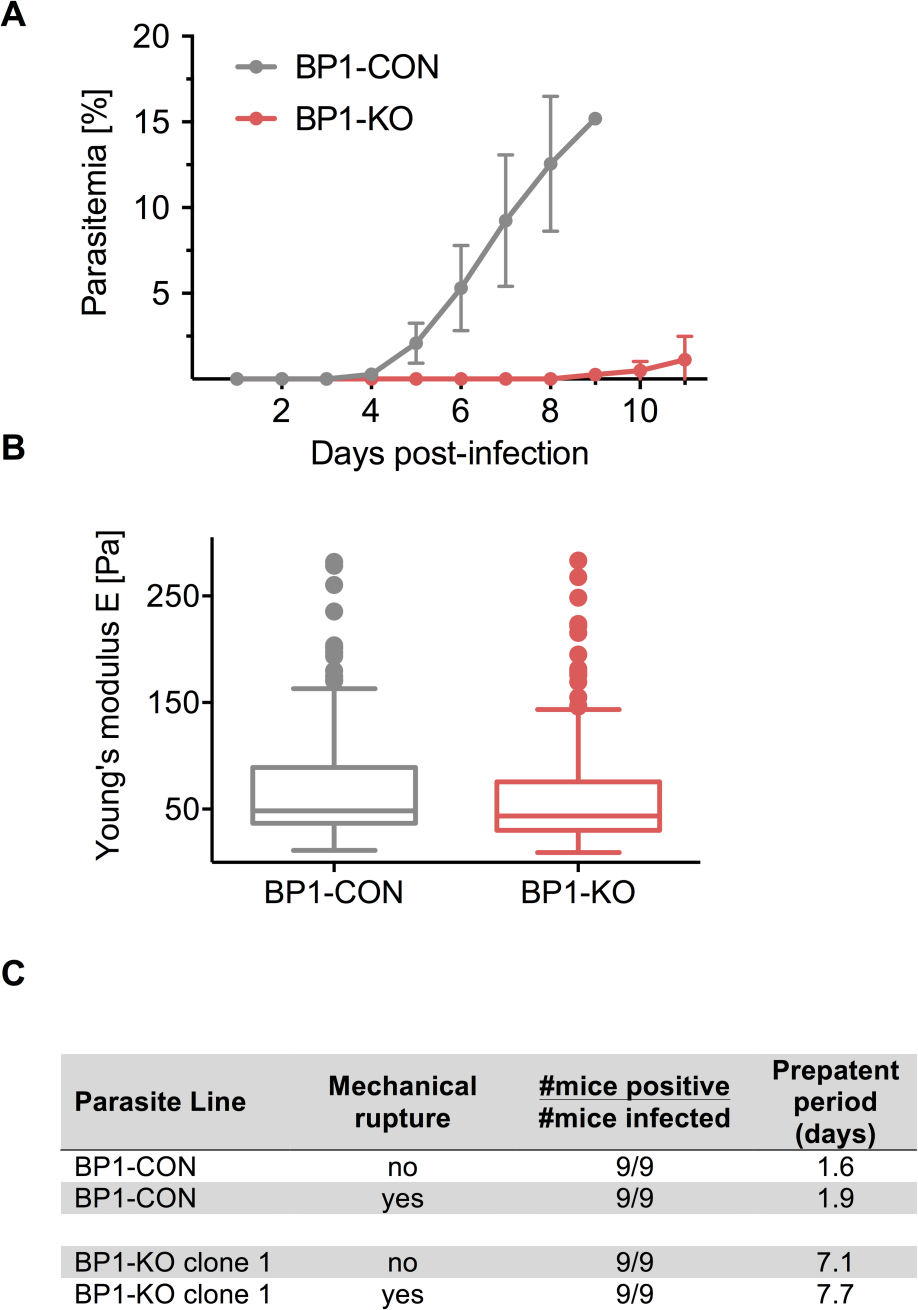
*Berghepain-1* knockout merosomes have reduced infectivity. **A. Detection of blood stage parasitemia following injection of merosomes.** Five merosomes of BP1-KO or BP1-CON parasites were injected i.v. per mouse and parasitemia was monitored daily for 11 days. Results from two representative experiments are shown and the average daily parasitemia ± SD of 5 mice infected with BP1-CON and 6 mice infected with BP1-KO clone 2 are plotted. **B. Atomic force microscopy of the merosome membrane.** Merosomes of BP1-CON and BP1-KO parasites were fixed and subject to AFM to measure Young’s modulus (E). Cell elasticity was measured on one point of each cell adhered to a poly-L-lysine-coated glass slide with 5 force-distance curves per cell. Data is displayed in a Tukey box and whisker plot, with a horizontal line representing the median and individually plotted outlier values. Shown are pooled values of seven independent experiments, four performed with BP1-KO clone 1 and three with BP1-KO clone 2, corresponding to approximately 195 cells for each clone. **C. Detection of blood stage parasitemia following injection of unruptured or mechanically ruptured merosomes.** 5000 ruptured or unruptured merosomes of BP1-KO or BP1-CON parasites were injected i.v. per Swiss Webster mouse. Average prepatent period of BP1-CON and BP1-KO clone 1 from two independent experiments performed are shown.

To test whether the BP1-KO merosomes produce infectious hepatic merozoites, we collected merosomes from *in vitro* cultures of BP1-CON and BP1-KO parasites, 60–65 h post-infection of HepG2 cells. Upon i.v. injection of five BP1-CON merosomes, mice became positive for blood stage parasites on day 4 after inoculation. In contrast, mice injected with BP1-KO exhibited a significant delay in prepatent period, with mice developing detectable parasitemia on day 9 after injection (Fig 4A).

Given that egress of *Plasmodium* blood-, liver- and mosquito-stages relies on protease activity [42] one possible explanation for this delay is that berghepain-1 may participate in the rupture of the merosome membrane and subsequent egress of the hepatic merozoites. Since infection by *Plasmodium* is known to alter the stiffness of hepatocyes, reducing their deformability [43], we hypothesized that the delay in prepatent period of BP1-KO parasites after injection of merosomes could be due to an altered elasticity of the merosome membrane, which would change its ability to rupture. To investigate this, we tested the elasticity of the membrane surrounding BP1-CON and BP1-KO merosomes by atomic force microscopy (AFM). No significant difference in merosome rigidity, represented by the Young’s modulus, a mechanical property of elastic solid materials [43], was found between populations of BP1-KO clone 1 and clone 2 and BP1-CON merosomes (Fig 4B), suggesting normal elasticity of the merosome membrane of BP1-KO parasites.

To further investigate whether reduced infectivity of BP1-KO merosomes was due to impaired merozoite release from the merosomes, we injected 5000 unruptured or mechanically ruptured merosomes into mice. Merosomes were mechanically disrupted with 10 strokes through a 30 gauge needle and immediately injected i.v. into mice. Microscopy of both BP1-CON and BP1-KO merosomes subject to this procedure confirmed that merozoites from 98% of merosomes were released. Upon injection of 5000 ruptured merosomes of the control parasite BP1-CON, parasites were detectable by Giemsa-stained blood smear after an average of 1.9 days (Fig 4C). In contrast, injection of the same number of ruptured merosomes of the BP1-KO parasite resulted in detectable blood stage parasitemia at an average of 7.7 days after injection, similar to the prepatent period of unruptured BP1-KO merosomes (Fig 4C). Thus, BP1-KO hepatic merozoites, when mechanically released from merosomes, have the same impairment in their ability to establish a blood stage infection as their unruptured counterparts. These data demonstrate that *berghepain-1* knockout hepatic merozoites are not adequately primed for erythrocyte invasion, and suggest a critical role for berghepain-1 in preparing hepatic merozoites for the successful infection of red blood cells either during the development of hepatic merozoites within the EEF or at the time of merozoite invasion of the red blood cell, or both.

### Attenuated growth and lethality of *berghepain-1* knockout blood stage parasites

**Fig 5:**
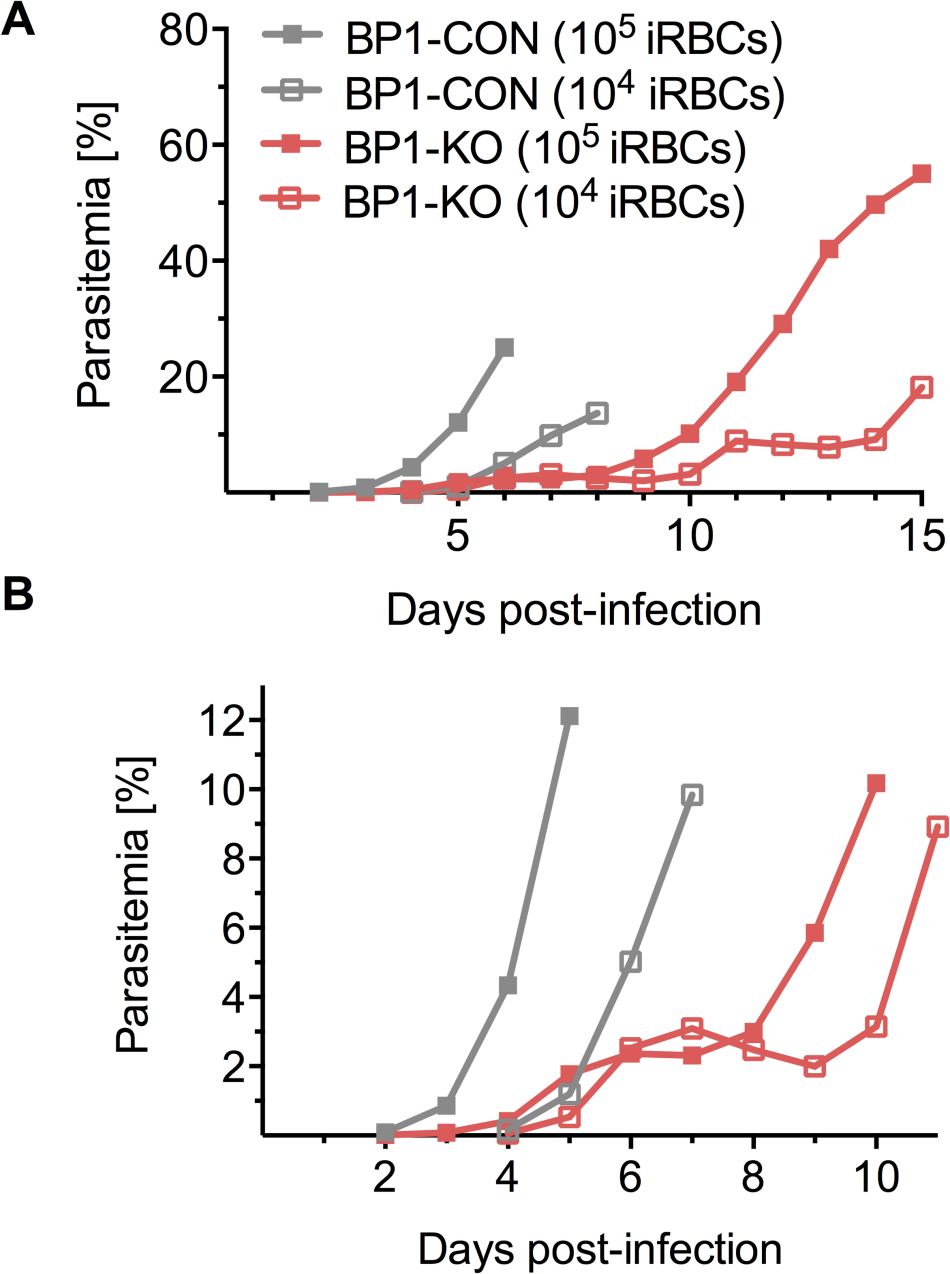
Blood stage growth of *berghepain-1* knockout parasites in mice. 10^4^ or 10^5^ red blood cells infected with BP1-CON or BP1-KO parasites were injected i.v. into Swiss Webster mice. **A.** Parasitemia was monitored daily and the average parasitemias of 5 mice per group is plotted. **B.** Data from days 1 through 10 shown in panel A, with an expanded Y-axis to highlight the differences in parasitemia in the early stage of infection. Results of a representative experiment, from a total of 2 experiments, using two different doses of BP1-KO clone 1, with a total of 5 mice per group, are shown.

Given the previous finding that falcipain-1 functions during blood stage merozoite invasion of erythrocytes [23], and our current finding that berghepain-1, the ortholog of falcipain-1, likely functions during hepatic merozoite infection of erythrocytes, we set out to characterize the *berghepain-1* knockout parasite in the blood stage. After infection with *P*. *berghei* ANKA blood stage parasites, parasitemia of susceptible mice typically rises rapidly and mice die within 7–8 days of experimental cerebral malaria, a syndrome characterized by inflammation in the brain and other organs [44–46]. We compared the growth of BP1-CON and BP1-KO parasites by inoculating equal numbers of infected red blood cells i.v. into Swiss Webster mice and monitoring parasitemia and survival of infected mice (Fig 5). All BP1-CON infected mice showed a rapid increase in parasitemia and death by day 8 post-infection. In contrast, in BP1-KO-infected mice, parasitemia initially increased but then plateaued for a few days (Fig 5B), after which it began to rise more rapidly. Mice inoculated with BP1-KO parasites died between days 14 and 19 post infection with high parasitemias. This is consistent with previous studies demonstrating that attenuation of *P*. *berghei* ANKA parasites or manipulation of the host immune response, prevents death from severe malaria [47–51]. Since the mice do not die an early death, the parasites continue to grow until high parasitemias ultimately kill the animal, likely from severe anemia. These data suggest that in addition to the role in priming hepatic merozoites for invasion, berghepain-1 has functions in the erythrocytic stage of the life cycle, consistent with a previous study which found a reduced blood stage growth rate for *P*. *berghei* berghepain-1 knockout parasites [13].

### Blood stage phenotype of *berghepain-1* knockout parasites can be restored by pretreating mice with phenylhydrazine

**Fig 6:**
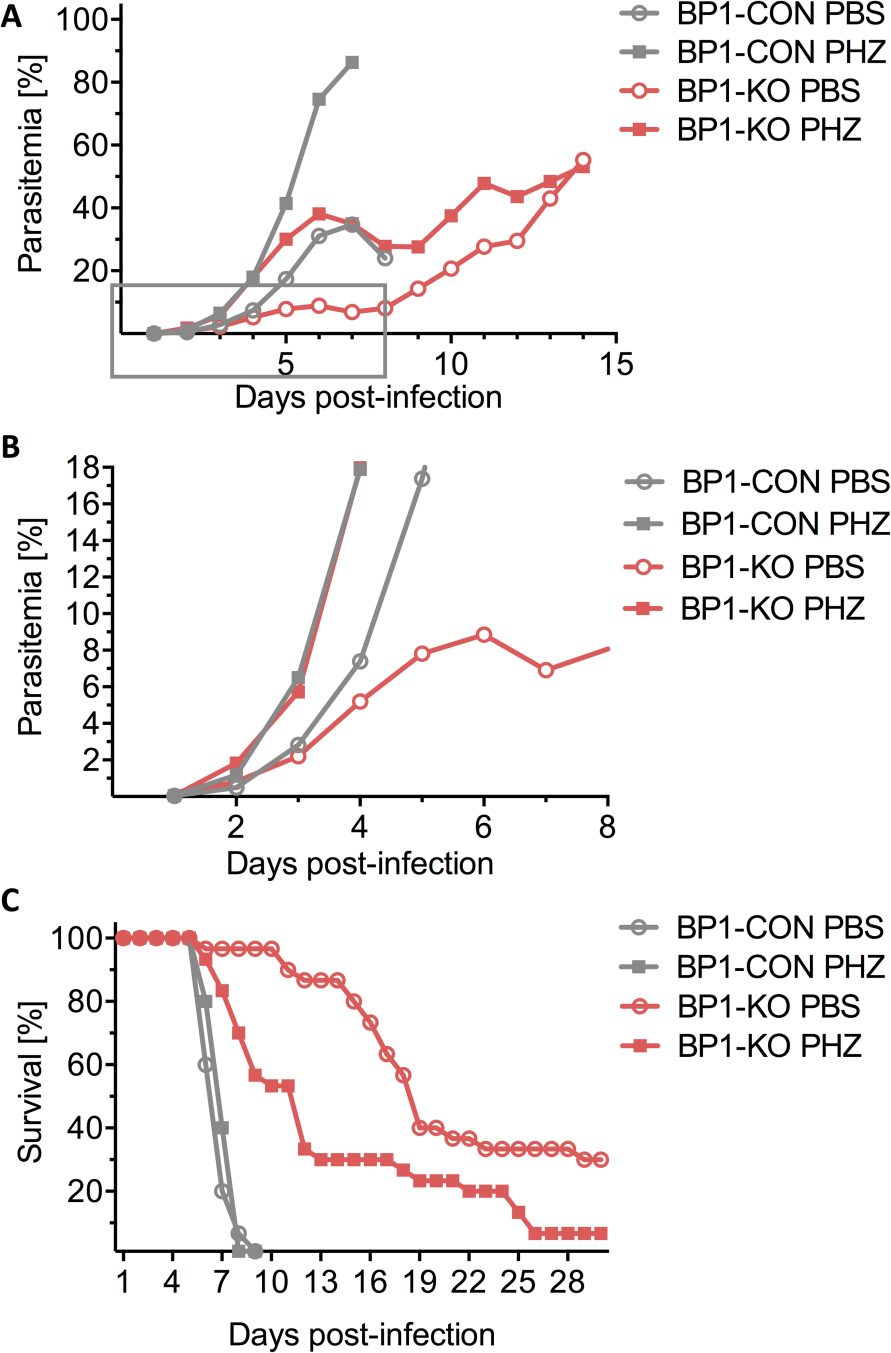
Blood stage growth of *berghepain-1* knockout parasites in phenylhydrazine (PHZ)-treated mice. Swiss Webster mice were pre-treated with PHZ (squares) or PBS (circles) with the last dose administered 3 days prior to i.v. injection of 2x10^6^ erythrocytes infected with BP1-CON or BP1-KO parasites. Pooled results from three biological replicates are shown, two using BP1-KO clone 1 and one using BP1-KO clone 2. **A.** Parasitemia was monitored daily and the average parasitemia of all surviving mice is plotted. **B.** The graph displays the data from days 1 through 8 (boxed region of panel A), to highlight differences in parasitemia in the early stage of infection. **C.** Survival curves of PHZ- and PBS-treated mice after infection with BP1-CON and BP1-KO parasites. Mice were monitored for 30 days and the average survival rate of infected mice is plotted.

The observed lag in parasite growth of BP1-KO asexual stages was reminiscent of the growth pattern observed in the non-lethal strains of *P*. *berghei* and *P*. *yoelii*, which have a marked preference for invading reticulocytes [52–54], young erythrocytes newly released from the bone marrow, which account for 1–3% of circulating erythrocytes in a non-anemic animal. For parasites with a reticulocyte preference, the plateau in parasitemia during the acute phase of infection reflects the depletion of available reticulocytes. This is followed by a rise in parasitemia as a consequence of the ensuing anemia, which induces a reticulocytemia [52–54]. We hypothesized that BP1-KO parasites are restricted to reticulocytes in manner similar to the non-lethal rodent malaria parasites. To test this experimentally, we induced a transient reticulocytemia in mice by pretreatment with the hemolytic agent phenylhydrazine (PHZ) [54]. Treated mice had more than 48% reticulocytes compared to 1% - 4% reticulocytes in PBS-treated control mice. Infecting PHZ-treated mice with BP1-KO parasites resulted in a rapid increase in parasitemia comparable to BP1-CON during the early stages of infection, achieving high parasite burdens and eliminating the plateau phase observed in PBS-treated mice infected with BP1-KO parasites (Fig 6A and 6B). Nonetheless, the effect of PHZ is temporary and by day 9 post-treatment (day 6 of infection), reticuolocyte counts return to baseline [54,55] and parasitemia once again plateaus. Of note, the parasitemia of PHZ-treated mice infected with BP1-CON parasites also rose more rapidly than in PBS-treated mice, likely due to a reticulocyte preference of wild-type *P*. *berghei* ANKA parasites. Furthermore, lethality, which is reduced in the BP1-KO parasite, is enhanced by PHZ-treatment: while only 15% of PBS-treated mice infected with BP1-KO had died by day 14, upon PHZ-treatment, 70% mice infected with BP1-KO parasites had died by day 14 (Fig 6C). These data demonstrate that the growth delay and lethality of BP1-KO parasites can be restored by increasing the reticulocytes available for invasion, suggesting that berghepain-1 is involved in erythrocyte tropism, specifically in mediating infection of mature erythrocytes.

### Berghepain-1 is critical for normocyte invasion

**Fig 7:**
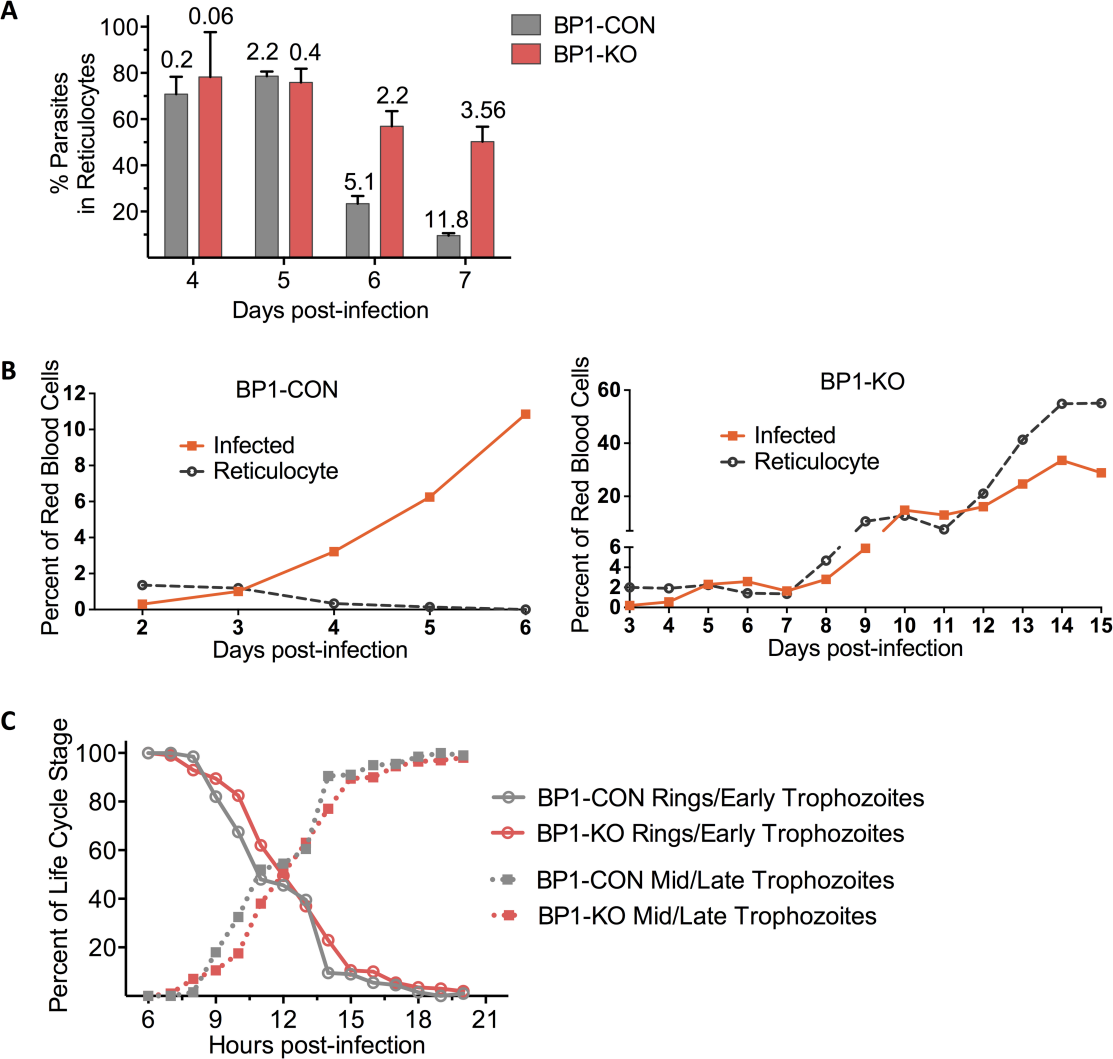
Berghepain-1 is important for normocyte invasion by blood stage parasites. **A. Reticulocyte preference of *berghepain-1* knockout parasites.** Swiss Webster mice were infected with 10^4^ purified BP1-CON and BP1-KO blood stage schizonts and infected normocytes and reticulocytes were counted starting 4 days after infection. Shown is the percentage of parasites found in reticulocytes on days 4–7 post infection. Each bar shows the mean plus standard deviation of 5 mice (BP1-CON) and 10 mice (BP1-KO), the latter being pooled data from BP1-KO clone 1 (5 mice) and BP1-KO clone 2 (5 mice). Mean total parasitemia values are displayed above the individual columns. **B. Growth of BP1-KO but not BP1-CON blood stage parasites follow the reticulocyte count.** Swiss Webster mice were injected i.v. with 2x10^5^ BP1-KO or BP1-CON infected erythrocytes. Parasitemia and reticulocytemia were monitored daily by Giemsa-stained blood smears and the average parasitemia of all surviving mice is plotted. n = 4 mice per group. **C**. **Normal cell cycle length of *berghepain-1* knockout parasites.** Infections in Swiss Webster mice were started by i.v. injection of 10^8^ BP1-KO or BP1-CON synchronized blood stage schizonts. Parasitemia was monitored hourly, counting ring stages and early trophozoites, and mid to late trophozoites. Percentage of each parasite life cycle stage is plotted as percent of total parasitemia and pooled results from two experiments using BP1-KO clone 1 are shown.

To further characterize the reticulocyte preference of *berghepain-1* knockout parasites, we inoculated 10,000 synchronized BP1-CON and BP1-KO blood stage schizont parasites and counted the number of parasites developing in reticulocytes versus normocytes from days 4 to 7 post infection. Infected reticulocytes were identified by simultaneous staining of blood smears with Giemsa-stain and a stain for reticulin, which is specific for reticulocytes [56]. Parasite infectivity for each of the two erythrocyte populations was determined by expressing the number of infected reticulocytes as a percentage of total infected red cells over days 4 to 7 post infection (Fig 7A). While BP1-CON parasites had a preference for reticulocytes at days 4 and 5, with over 60% of parasites invading reticulocytes, this percentage dropped to below 20% as the total parasitemia increased (Fig 7A). During early stages of infection, BP1-KO parasites behaved similarly, with 75% of BP1-KO parasites developing in reticulocytes at day 4, however in contrast to the BP1-CON parasite, this preference only marginally dropped over the following days. These data suggest that reticulocytes are the preferred target cells for both control and BP1-KO *P*. *berghei* parasites. However, as reticulocytes are consumed by the infection, only control parasites are readily able to invade normocytes, supporting a role for berghepain-1 in infection of mature erythrocytes.

To further confirm that the growth pattern of BP1-KO parasites was due to their reticulocyte restriction, we inoculated BP1-KO and BP1-CON infected red blood cells i.v. into Swiss Webster mice and monitored both parasitemia and reticulocyte counts over time. As expected, control parasites grew rapidly despite low reticulocyte numbers and quickly killed the mice (Fig 7B). In contrast, BP1-KO parasite growth followed the expansion of the reticulocyte pool, initially growing to parasitemias of ~ 2 to 3%, then plateauing and only increasing after the induction of a reticulocytosis (Fig 7B).

Since an overall longer cell cycle can also produce a slow-growing phenotype, we set out to investigate whether the slow growth of *berghepain-1* knockout blood stage parasites could be due to a change in length of cell cycle. Synchronized blood stage schizonts were injected into a mouse and hourly Giemsa-stained blood smears were used to monitor the transition from G1 to S-phase, which occurs between early and mid-trophozoite stage [57]. Using percent of total parasites that were rings/early trophozoites versus mid/late trophozoites as a readout for cell cycle duration, we found that BP1-KO parasites cell cycle mirrored that of BP1-CON parasites (Fig 7C). It should be noted that the schizont stage of *P*. *berghei* parasites adhere to endothelium and are not circulating; thus, after 20 h, numbers of circulating parasites decreased as mature trophozoites developed into schizonts. Our data suggest that the slower growth in BP1-KO parasites is due to a deficiency in infecting erythrocytes rather than slower development.

### Key role of berghepain-1 for hepatic merozoite infectivity

Our data demonstrate that deletion of berghepain-1 gives rise to a phenotype in both blood stage and hepatic stage merozoites. We hypothesized that the role of berghepain-1 in these two distinct populations of merozoites is not equivalent since BP1-KO merosome inoculation results in a prepatent period delay of 5 days compared to control parasites (Fig 4) whereas BP1-KO blood stage parasites are less attenuated (Figs 5–7). To directly compare the infectivity of BP1-KO hepatic and blood stage merozoites, we compared the onset of detectable parasitemia in Swiss Webster mice inoculated with synchronized schizonts and merosomes. Mice were injected i.v. with 1,000 or 10,000 purified blood stage schizonts derived from *in vitro* overnight culture of blood stage BP1-CON and BP1-KO parasites or with 1,000 BP1-CON and BP1-KO hepatic merosomes isolated from *in vitro* liver stage cultures. While schizonts contain between 10 to 14 individual merozoites [58], merosomes are more variable, harboring between 100 and 1000 hepatic merozoites [3]. Mice injected with 1,000 or 10,000 BP1-CON blood stage schizonts developed detectable parasitemia by day 4 and 3, respectively. After injection of the same number of BP1-KO blood stage schizonts, a delay of 0.6 days and 1 day, respectively, was seen compared to BP1-CON (Table 2). This was in stark contrast to mice injected with merosomes: while injection of BP1-CON merosomes led to detectable blood stage parasitemia within one day, we did not detect parasites until 5.6 days after BP1-KO merosome inoculation. Thus, the delay to patency of BP1-KO merosomes was greater by ~ 4 days compared to the delay observed with BP1-KO blood stage schizonts.

**Table 2:** Liver and blood stage merozoite infectivity of *berghepain-1* knockout parasites. Swiss Webster mice were injected i.v. with purified blood stage schizonts or merosomes of BP1-CON and BP1-KO clone 1 parasites and parasitemia was monitored daily. In the last set of experiments, mice were pre-treated with phenylhydrazine (PHZ) prior to inoculation of BP1-CON and BP1-KO merosomes.

Parasite	Dose	Infectivity (#mice infected #mice inoculated)	Prepatent period (days)
BP1-CON	1,000 schizonts	5/5	4.00
BP1-KO	1,000 schizonts	5/5	4.60
BP1-CON	10,000 schizonts	5/5	3.00
BP1-KO	10,000 schizonts	5/5	4.00
BP1-CON	1,000 merosomes	20/20	1.00
BP1-KO	1,000 merosomes	20/20	5.65
BP1-CON	1,000 merosomes	10/10 PHZ-treated mice	1.00
BP1-KO	1,000 merosomes	10/10 PHZ-treated mice	4

Since the delay in prepatent period after inoculation of BP1-KO hepatic merozoites may in part result from reduced invasion capacity of the erythrocytic merozoites in the ensuing blood stage infection, we attempted to restore growth by inoculating merosomes into mice pretreated with phenylhydrazine. This improved the infectivity of BP1-KO merosomes by ~ 1.5 days, suggesting that the patency delay of BP1-KO merosomes reflects the combined delay in both populations of merozoites. However, in contrast to blood stage merozoites, reticulocytosis does not fully restore BPI-KO merozoite infectivity. Overall, these experiments demonstrate that hepatic merozoites of the berghepain knockout parasite are significantly more impaired than erythrocytic merozoites and highlight that while both hepatic and erythrocytic merozoites invade red blood cells, clear differences exist between the merozoites released from the liver and those released from infected red blood cells.

### Berghepain-1 is expressed in both blood and liver stage parasites

**Fig 8:**
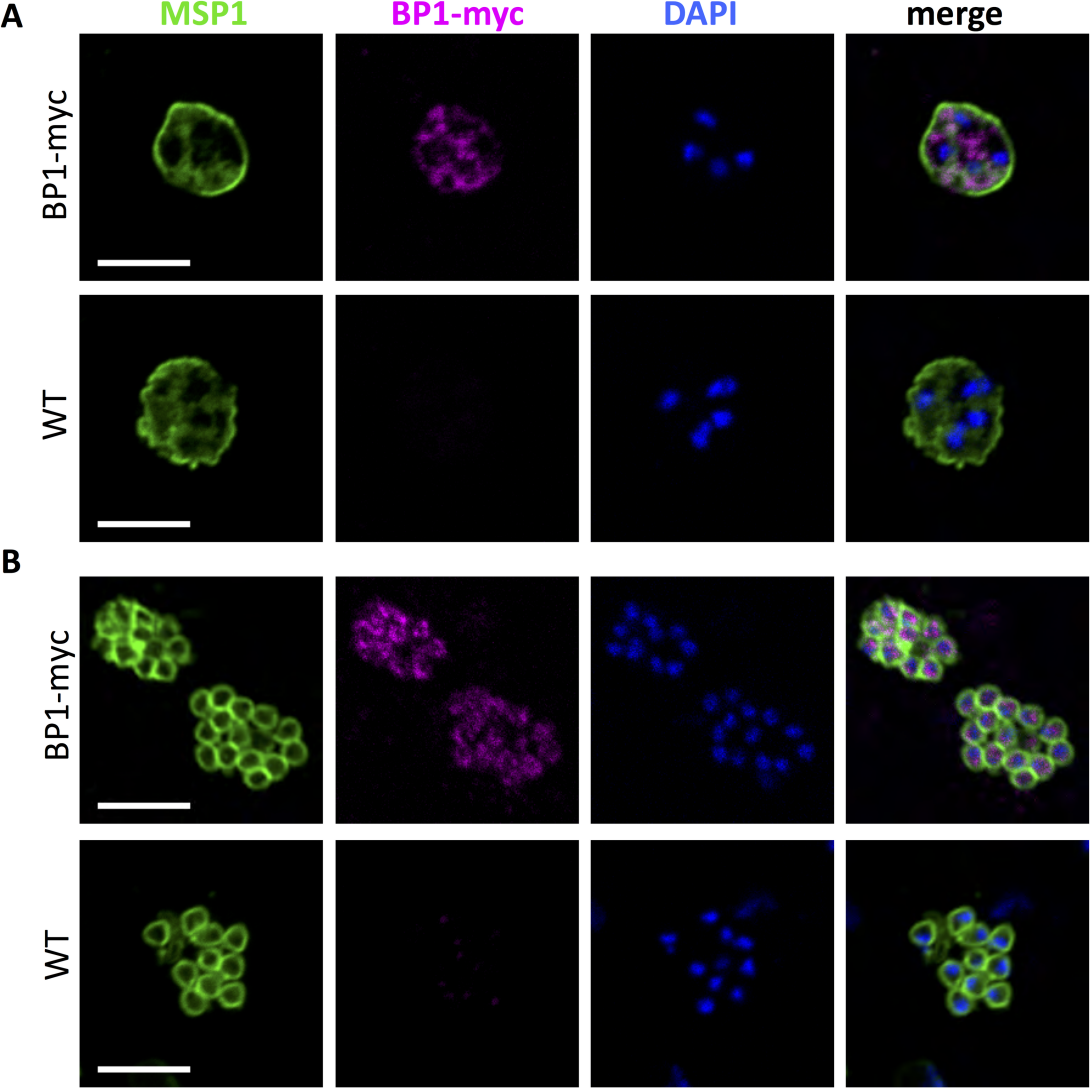
Expression of berghepain-1 in blood stage parasites. Early (panel A) and late (panel B) blood stage schizonts of transgenic berghepain-1-myc parasites and WT parasites stained with anti-c-Myc (magenta) to visualize berghepain-1-myc, and anti-MSP1 (green) to visualize the merozoite membrane. Parasite DNA was stained with DAPI (blue). Scale bars: 5 μm.

**Fig 9:**
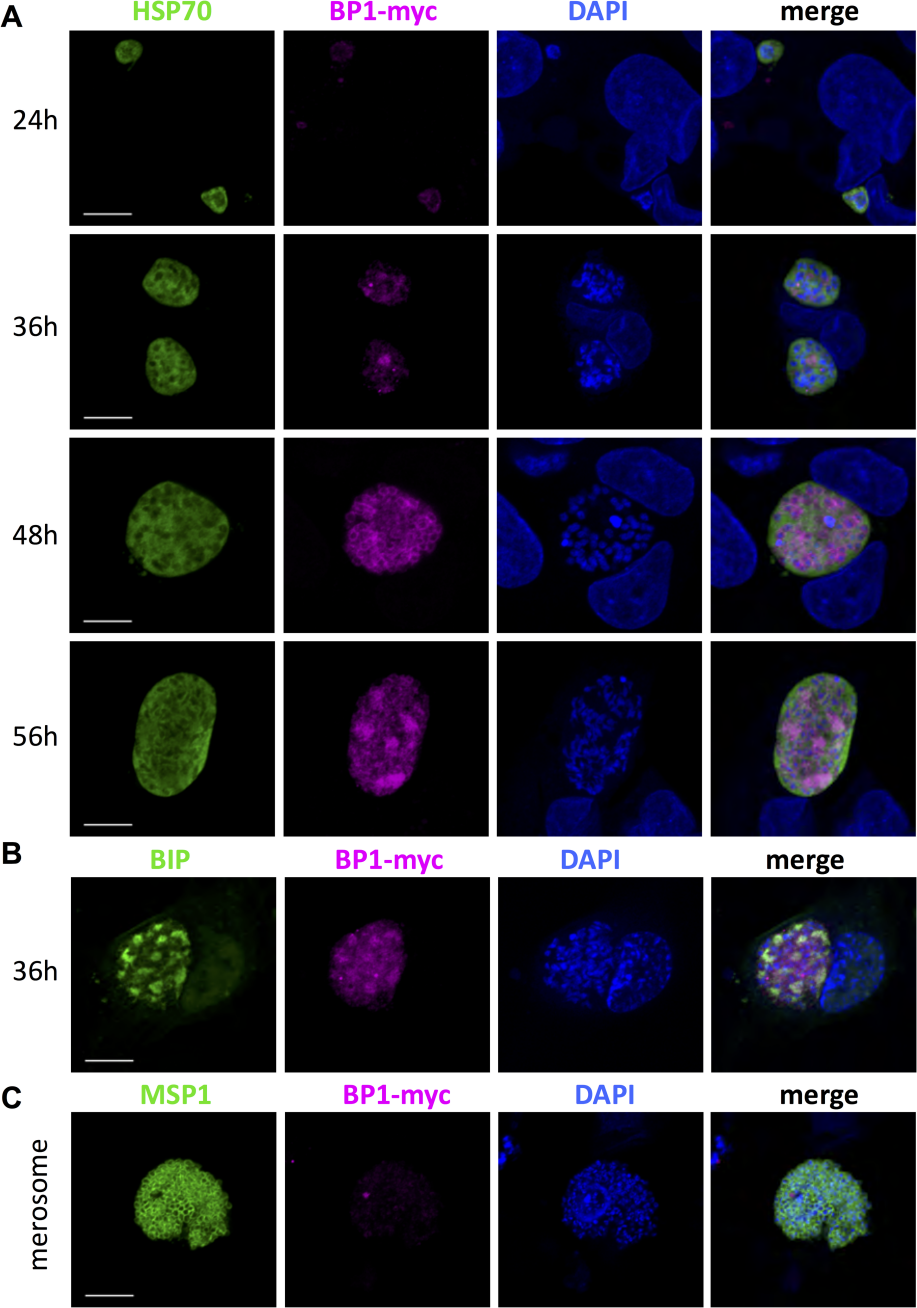
Expression of berghepain-1 in liver stage parasites. HepG2 cells infected with BP1-myc parasites, and merosomes collected from these cultures, were fixed at the indicated timepoints and probed with anti-c-myc antibodies (magenta) to localize berghepain-1, and co-localized with antibodies against **A.** the cytosolic marker heat-shock protein 70 (HSP70), **B.** the ER chaperone BIP (green) and **C.** the membrane maker merozoite surface protein 1 (MSP-1). BP1-myc expression was low 24 h after invasion, but seen in early schizonts after 36 h of development. Host and parasite DNA were stained with DAPI (blue). Scale bars: 10 μm.

To investigate the timing and localization of berghepain-1 expression, we generated a parasite line in which the endogenous *berghepain-1* gene was fused to a triple myc tag, a short sequence derived from the *c-myc* gene (S5 Fig). Using this line, we investigated whether berghepain-1 is expressed at the protein level in blood stage parasites, performing immunofluorescence microscopy of early and late blood stage schizonts. As shown in Fig 8, berghepain-1-myc is expressed in early schizonts and the staining localizes to the individual merozoites in segmented mature schizonts. We then investigated berghepain-1 expression during liver stage development. Immunofluorescence microscopy of HepG2 cells infected with myc-tagged berghepain-1 parasites showed low levels of berghepain-1-myc expression at 24 h post infection, which increased at 36 h in late hepatic trophozoite stages (Fig 9A). At 48 h post infection, berghepain-1-myc surrounded the individual nuclei and this perinuclear pattern was still present at 56 h (Fig 9A). At 33 h post infection, berghepain-1-myc was also found in larger sub-compartments of the parasites, which co-localized with the staining of the ER marker BiP [59,60] (Fig 9B). Berghepain-1-myc did not co-localize with cytosolic marker HSP70 [61], or the membrane marker MSP1 (Fig 9A and 9C) or with CSP (S6 Fig). The specificity of the anti-c-myc staining is demonstrated by the lack of staining of liver stages of the parental control line (S7 Fig). Though these data demonstrate that berghepain-1 is expressed during liver stage development, we did not, however, detect expression of berghepain-1-myc in merosomes, the stage that is attenuated in berghepain-1 deletion mutants (Fig 9C). Thus, it is possible that berghepain-1 functions prior to the formation of merosomes, priming merozoites that will then be packaged into merosomes for exit from the liver. We cannot, however, eliminate a role for berghepain-1 in hepatic merozoites as it’s possible that expression levels in hepatic merozoites are too low to be detected by our methodology. The latter possibility is consistent with expression data of other proteases whose low abundance makes it difficult to detect [62,63].

## Discussion

The function of falcipain-1, the most highly conserved member of the falcipain family of proteases, has been the subject of some controversy. While an inhibitor of falcipain-1 blocked erythrocyte invasion by merozoites [23], two independent studies showed that falcipain-1 disruption did not affect growth of blood stage parasites [29,30]. Since the rodent model affords a more in-depth analysis of protein function across all life cycle stages of *Plasmodium*, we disrupted *berghepain-1*, the falcipain-1 ortholog of the rodent parasite *P*. *berghei*, in an attempt to better understand the role of this protease. Our study revealed that berghepain-1 has a role in erythrocyte infection by both hepatic and erythrocytic merozoites. Furthermore, the impact of berghepain-1 deletion is significantly more pronounced in hepatic merozoites, indicating that hepatic merozoites are not identical to their blood stage counterparts.

An important role for berghepain-1 in erythrocyte infectivity by blood stage rodent malaria parasites is supported by several lines of evidence: Berghepain-1 knockouts have a growth delay, consistent with previous work [13], and reduced lethality, which can be restored, at least temporarily, by increasing the pool of young erythrocytes. The reticulocyte tropism of berghepain-1 knockout parasites was further confirmed by reticulin staining of infected cells, and analysis of cell cycle duration showed normal development of the mutant parasite following invasion, indicating that the defect in berghepain-1 knockout merozoites is specific to one or more steps in the entry process rather than growth. Indeed, previous work showed that asexual blood stages of berghepain-1 deletion mutants produce wild-type levels of hemozoin, suggesting that unlike berghepain-2, the function of berghepain-1 is not associated with hemoglobin digestion [13]. Our findings are supported by a previous study demonstrating that an inhibitor of falcipain-1 impacts erythrocyte invasion by *P*. *falciparum* and the localization of falcipain-1 to the apical end of merozoites [23]. However, two subsequent studies showed that deletion of falcipain-1 did not result in a blood stage growth phenotype [29,30], raising the possibility that there are essential differences between the host cell invasion pathways used by rodent and human parasites. Another possibility is that the selective pressure generated by many rounds of replication during *in vitro* culture of *P*. *falciparum* could select for parasites that are able to compensate invasion defects. Indeed, *P*. *falciparum* merozoites can invade erythrocytes using multiple pathways and some of these may not rely on the activity of falcipain-1. This is supported by our observation that berghepain-1 knockout parasites are not dramatically inhibited in erythrocytic stage growth, indicating that in the rodent parasites as well, alternate invasion pathways are utilized by the BP1-KO parasites. Thus, taken together these data raise the possibility that falcipain-1 and its orthologs have a conserved role across species.

Given the reticulocyte tropism of the BP1-KO parasite, we hypothesize that berghepain-1 is involved in infection of mature erythrocytes, possibly by cleaving a parasite ligand required for this process. This could function in initial adhesion to the host cell or in the invasion process, either of which would be consistent with our data. Supporting this hypothesis is evidence that cathepsin L, a falcipain-like protease in the related apicomplexan parasite *Toxoplasma gondii*, was found to proteolytically mature adhesins as they traffic to micronemes, the specialized secretory organelles whose regulated secretion is essential for invasion [64]. Though the substrate(s) for berghepain-1 remain unknown, possible candidates include the rodent malaria 235 kDa rhoptry proteins [65,66], members of the reticulocyte-binding-like (RBL) protein family found in all *Plasmodium* species and known to be involved in erythrocyte invasion. In *P*. *yoelii*, Py235 proteins influence host erythrocyte preference and are associated with virulence, with more virulent parasites invading a wider range of erythrocytes [37,67,68]. Interestingly, distinct subsets of Py235 proteins are expressed in liver and blood stage parasites [69]. Future work involving mass spectrometric approaches that probe for potential cognate substrates of berghepain-1 will shed additional light on the function of this protease.

We also found a critical role for berghepain-1 in the pre-erythrocytic stage of infection. The pronounced delay in blood stage infection after sporozoite inoculation suggested that berghepain-1 functions at one or more steps between sporozoite localization to the liver and initiation of blood stage infection. Experiments designed to test each stage of this process revealed that injection of berghepain-1 knockout merosomes could replicate the pronounced delay in blood stage infection after berghepain-1 knockout sporozoite inoculation. Additional experiments with mechanically ruptured merosomes pinpointed the defect to a decreased infectivity of merozoites arising from mature liver stage parasites. Given the expression of BP1-myc during EEF development, this suggests a role for berghepain-1 in preparing hepatic merozoites for infection of red blood cells.

Intriguingly, while these two distinct merozoite populations appear morphologically identical and are functionally similar in that both must invade red blood cells, we observed a more significant attenuation of BKO-1 hepatic merozoites compared to their blood stage counterparts. Though we do not yet understand how the same protease differentially impacts these distinct merozoite populations, there are two possible scenarios. One possibility is that the same ligand is processed in blood stage and hepatic merozoites, with this event having a more critical role in hepatic merozoite infectivity. Alternatively, berghepain-1 could have a different role, possibly processing a different substrate, in each of these merozoite populations. Studies with endogenously-tagged berghepain-1 showed that it localizes to merozoites of blood and liver stage schizonts, but is not found in merosomes. These localization data are consistent with either possibility since parasite egress from the mother cell differs in these two merozoite populations. In the blood stage, PV rupture and erythrocyte membrane rupture occur in rapid succession whereas in the liver, the formation of merosomes is an additional step, occurring after PV rupture, and enabling hepatic merozoites to exit the liver sinusoid. Thus, if berghepain-1 acts at some point prior to PV rupture in both merozoite populations, it is not surprising that the merosomes, with already primed merozoites, contain little berghepain-1. Unfortunately, the small amount of hepatic merozoite material that can be collected combined with the lack of an *in vitro* infectivity assay for hepatic merozoites, have made it difficult to more precisely determine the hepatic merozoite defect. Future work focusing on identification of the berghepain-1 substrate(s) will be critical to elucidating its role in hepatic and blood stage merozoite infectivity.

Little is known about whether there are finer-scale differences between blood stage and hepatic merozoites, with only one previous study addressing this topic. This elegant work demonstrated that different Py235 family members are expressed in hepatic versus erythrocytic merozoites [69]. Based on their data, these authors suggested that hepatic and blood stage merozoites differentially rely on distinct invasion pathways, a hypothesis that is supported by our data. This makes sense in light of the different biological niches of each merozoite population. Given the bottleneck of sporozoite transmission, hepatic merozoites likely originate from 1 to 5 infected hepatocytes and as a result, their numbers are 3 to 5 logs lower than their blood stage counterparts [70]. Despite their low numbers, it is essential that hepatic merozoites succeed in invading erythrocytes for if they fail, gametocytes will not be produced for transmission to the mosquito. This is in contrast to blood stage merozoites which are present in large numbers and thus risk killing the host, a scenario which would also jeopardize transmission to the mosquito. Thus, while it is imperative for hepatic merozoites to maximize their infectivity for erythrocytes, blood stage merozoites must walk a line between maintaining infection and not killing the host. Therefore it is plausible that these two populations of merozoites differ in their invasion pathways in ways that are more complex than we can currently appreciate. Future work comparing liver and blood stage merozoites to better understand their differences will help inform the search for suitable drug targets for prophylactic or dual stage drug interventions.

## Materials and methods

### Ethics statement

All animal work was conducted in accordance with the recommendations by New York University and Johns Hopkins University Animal Care and Use Committees (ACUC), under the ACUC-approved protocols 110608, M011H467 and M014H363. All animal experiments performed at the LUMC were approved by the Animal Experiments Committee of the Leiden University Medical Center (12042). The Dutch Experiments on Animal Act were established under European guidelines (EU directive no. 86/609/EEC regarding the Protection of Animals used for Experimental and Other Scientific Purposes). All efforts were made to minimize suffering. Experiments were performed in male and/or female 4- to 6-week-old Swiss Webster or NMRI mice and C57BL/6 mice, purchased from Taconic and Charles River. Male Wistar-Kyoto rats were also used for transfection experiments.

### Generation of BP1-CON and BP1-KO parasite lines

Recombinant *P*. *berghei* BP1-CON and BP1-KO parasites were generated by double homologous recombination in which the native *berghepain-1* locus was replaced with a selection cassette (BP1-KO) or a wildtype copy of the *berghepain-1* with the selection cassette (BP1-CON). Targeting plasmid pBP1-KO was generated by flanking the human dihydrofolate reductase (hDHFR) cassette in plasmid pDEF-hDHFR-flirte [71] with 1.6 kb of the *berghepain-1* 5’ UTR and 1.3 kb of *berghepain-1* 3’ UTR, both cloned from gDNA (S1 Fig). For the control construct pBP1-CON, 1.84 kb of the *berghepain-1* 5’ UTR, 1.56 kb of the *berghepain-1* ORF and 1.33 kb of the *berghepain-1* 3’ UTR were cloned from gDNA and inserted into pDEF-hDHFR-flirte as outlined in S1 Fig. Transfection was performed using plasmid digested with EcoR1 to liberate the DNA fragment, containing sequence from the 5’ and 3’ UTRs of *berghepain-1*, to drive double homologous recombination. *P*. *berghei* ANKA parasites clone 507cl1 [31], were electroporated with 5 μg of digested plasmid DNA, injected into mice, selected with pyrimethamine and cloned by limiting dilution in mice, following standard procedures [72].

### Generation of berghepain-1-myc expressing parasites

The reporter line, *Pb*GFP-Luc_schz_ (line 1037cl1; www.pberghei.eu mutant RMgm-32;) was used to generate the transgenic berghepain-1-myc line. In this line, the *gfp-luc* expression cassette is stably integrated into the *230p* locus without introduction of a drug-selectable marker and is under the control of the blood stage schizont-specific *ama1* promoter [50]. The *berghepain-1* ORF (without its stop codon) was PCR-amplified from wild type *P*. *berghei* ANKA genomic DNA with primer sets L7424/L7425 (see S1 Table). This PCR product was digested with *Spe*I and *BamH*I, and C-terminally fused to a triple *c-myc* tag by ligation into the *Spe*I/*BamH*I digested vector pL1612, resulting in construct pL2018 (S5 Fig). Prior to transfection, pL2018 was linearized with *Afl*II. Transfection, selection and cloning of transgenic parasites with pyrimethamine were carried out as described previously [72], generating the transgenic line berghepain-1-myc (line 2338), expressing endogenously C-terminally tagged berghepain-1.

### Mosquito infection

*Anopheles stephensi* mosquitoes were reared using standard procedures and fed on Swiss-Webster mice infected with the indicated parasite line. On day 13 after infective blood meal, mosquitoes were dissected and the midguts were observed for oocyst counts using an upright Nikon E600 microscope with a phase contrast PlanApo 10x objective. For salivary gland sporozoite numbers, salivary glands were harvested on day 19 after infective blood meal from 20 mosquitoes and counted on a hemocytometer.

### Sporozoite gliding motility assay

Sporozoite gliding motility was assayed as previously described [25]. Glass 8-chambered Lab-tek wells (ThermoScientific) were coated with 10 μg/μl mAb 3D11, specific for the repeat region of the *P*. *berghei* circumsporozoite protein [73], in PBS overnight at 25°C. Salivary gland sporozoites in 3% BSA in Dulbecco's Modified Eagle Medium (DMEM) were added to each well and incubated for 1 h at 37°C. Wells were fixed in 4% paraformaldehyde and trails were visualized by staining with biotinylated mAb 3D11, followed by detection with strepativin conjugated to FITC (Amersham). Trails associated with sporozoites and the number of circles per trail were counted using fluorescence microscopy on an upright Nikon E600 and 40x objective.

### Sporozoite infectivity as determined by prepatent period

To examine sporozoite infectivity *in vivo*, 4- to 6-week-old Swiss Webster or C57BL/6 mice were inoculated i.v. with the indicated number of sporozoites in DMEM. The onset of blood stage infection was determined by daily observation of Giemsa-stained blood smears, beginning on day 3 after inoculation. For intradermal inoculation, mice were lightly anesthetized by intraperitoneal injection of ketamine/xylazine (35–100 μg ketamine/g body weight) and maintained at 37°C on a slide warmer. Sporozoites were injected into the ear pinna, in a total volume of 0.2 μl DMEM, with a Flexifill microsyringe (World Precision Instruments).

### Sporozoite infectivity as determined by quantification of liver stage parasite burden

To examine *in vivo* sporozoite development in the liver, 4- to 6-week-old Swiss Webster or C57BL/6 mice were inoculated i.v. with 10,000 sporozoites in 200 μl of DMEM. 40 h later, livers were harvested for total RNA isolation and infection was quantified using reverse transcription followed by real-time PCR, using primers that recognize *P*. *berghei*–specific sequences within the 18S rRNA, as outlined previously [74]. Copy number was ascertained by comparison with a plasmid standard curve.

### Parasite development in hepatocyte cultures

Cells of the human hepatoma cell line HepG2 (ATCC, HB-8065) were maintained in DMEM supplemented with 1 mM L-glutamine, 10% Fetal Calf Serum and 5 mg⁄mL penicillin/streptomycin (complete medium) at 37°C and 5% CO_2_, as previously described [75]. 2.5 x 10^5^ HepG2 cells per well were plated onto coverslips coated with collagen I (BD Biosciences #354236) and grown for 8–12 h in 24-well plates. Sporozoites were dissected in DMEM and 4–6 x 10^4^ sporozoites were added per well. After sporozoites were allowed to invade for 2 h at 37°C, free sporozoites were removed by washing with complete medium containing 5μg/mL Fungizone (Cellgro 30-003-CF) and 10X penicillin/streptomycin (wash medium), and then maintained in complete medium. Cells were washed twice per day with wash medium until the indicated timepoint, when they were fixed with 4% paraformaldehyde (PFA) and mounted. EEFs were observable due to their GFP-expression, and total EEFs per coverslip were manually counted. For immunofluorescence assays, EEFs were stained as outlined below.

### Merosome production and harvest

To quantify the formation of merosomes, HepG2 cells were grown at a density of 50,000 cells per well of a 24-well plate and infected as described above. At 50 h post infection, culture supernatant volume was reduced to 0.5 ml medium/well. Culture supernatant containing merosomes was collected between 60 and 65 h post infection using a pasteur pipette and counted using a hemocytometer. Merosomes (numbers depending on the experiment) were injected i.v. into Swiss Webster mice for prepatent period experiments. For mechanical rupture assays, 25 merosomes per μl in a total volume of 1 ml were sheared by 10 strokes through a 30 gauge needle using a 1 ml syringe and within 5 min were injected i.v. into mice. Samples from ruptured and control merosomes were fixed in 0.4% PFA and nuclei were stained with DAPI to allow microscopic analysis of rupture, which revealed that 98% of merosomes were ruptured by the procedure.

### Cell elasticity measurement on merosomes cells

For atomic force microscopy experiments, infection with BP1-CON and BP1-KO clones 1 and 2 was allowed to proceed in HepG2 cells until 65 h post infection, when merosomes were collected from the culture supernatant. Total medium from two infected wells of a 24-well plate was collected in a 1.5 ml tube and merosomes were allowed to settle for 15 min at room temperature. Medium was then carefully removed to leave ~30–50 μl containing the merosomes and 500 μl of 1% PFA was added to fix the merosomes, in order to stop their movement. After 3 min, 1 ml of PBS was added and merosomes were again allowed to settle for 15 min at room temperature. The paraformaldehyde solution was removed, leaving 30–50 μl and 150 μl DMEM was added to the merosomes. Nanoindentation experiments were carried out at 25°C using an atomic force microscope *NanoWizard II* (JPK Instruments, Berlin, Germany) mounted on the top of an Axiovert 200 inverted microscope (Carl Zeiss, Jena, Germany). Measurements were made using non-functionalized OMCL TR-400-type silicon nitride tips (Olympus, Japan). Tip spring constants were calibrated by the thermal fluctuation method, having a nominal value of 0.02 N/m. For cell contact, the distance between the cantilever and the cell was adjusted to maintain a maximum applied force of 800 pN before retraction. Data collection for each AFM force-distance cycle was performed at 1.5 Hz and with a z-displacement range of 8 μm. The acquired force curves were analyzed using JPK Image Processing v. 4.2.53, by the application of the Hertzian model, to obtain the cells Young’s modulus (E). The AFM probe was modeled as a quadratic pyramid, with a tip angle of 35° (half-angle to face) and a Poisson ratio of 0.50. For data analysis, multiple readouts of Young’s modulus from a single cell were averaged and the mean used to represent the value for that cell. Rare outlier values above 300 Pa were discarded, though statistical significance of variance between BP1-CON and BP1-KO populations did not change if they were included.

### Immunofluorescence assays of EEFs and merosomes

For IFAs of EEFs, wells with infected HepG2 were washed and fixed in 4% paraformaldehyde/PBS for 1 h at room temperature. For IFAs of merosomes, supernatants were collected and spun at 50x*g* onto poly-L-lysine-coated coverslips and fixed for 20 min with 4% PFA at room temperature. Both EEF and merosomes were permeabilized in methanol overnight at -20°C and blocked with 1% BSA/PBS for 1 h at room temperature before incubation with primary and secondary antibodies for 1 h each at room temperature. The following antibodies were used, diluted in 1% BSA/PBS: mouse anti-MSP 25.1 diluted 1:500 [37], polyclonal rabbit anti-UIS4 diluted 1:5000 [41], mouse anti-CSP at 1 μg/ml [clone 3D11; [73]], mouse anti-*Plasmodium* HSP-70 diluted 1:500 [clone 2E6; [61]], mouse anti-BiP diluted 1:200 [60], and rabbit anti-c-myc diluted 1:400 (C3956, Sigma). Secondary antibodies used were anti-mouse Alexa Fluor 488 conjugate (A11029, ThermoFisher) and anti-rabbit Alexa Fluor 594 conjugate (A11012, ThermoFisher), each diluted 1:500. Samples were preserved in Prolong Gold mounting medium containing DAPI (Life Technologies). Images for Figs 2 and 3 were acquired using an upright Nikon 90i fluorescence microscope and a 40x objective. Images for Fig 5, S5 and S6 Figs were acquired using a LSM700 laser scanning confocal microscope (Zeiss AxioObserver) with a 63x/1.4 PlanApo oil objective using Zen software.

### Immunofluorescene assays of blood stages

Thin blood smears were air-dried, fixed with 4% paraformaldehyde/PBS, permeabilized with 0.1% Triton X-100/PBS and blocked with 3% BSA/PBS before incubation with primary and secondary antibodies. Rabbit anti-c-myc antibody (C3956, Sigma) was diluted 1:400 and mouse anti-MSP 25.1 [37] was diluted 1:2000 in 1% BSA/PBS. Secondary detection was with anti-mouse Alexa Fluor 488 conjugate (A11029, ThermoFisher) and anti-rabbit Alexa Fluor 594 conjugate (A11012, ThermoFisher), each diluted 1:500. Samples were preserved in Prolong Gold mounting medium containing DAPI (Life Technologies) and imaged using a LSM700 laser scanning confocal microscope (Zeiss AxioObserver) with a 63x/1.4 PlanApo oil objective and images were acquired using Zen software.

### Phenylhydrazine treatment

Mice were treated with phenylhydrazine (PHZ; Sigma-Aldrich, P26252) dissolved in PBS pH 7.4, delivered intraperitoneally at 100 μg/g of body weight. Three total doses were given, administered every other day, and mice were injected with infected red blood cells three days after the final dose. To monitor reticulocyte numbers, blood smears were Giemsa-stained and reticulocytes, which stain blue due to residual RNA, were counted as a percentage of total erythrocytes.

### Schizont culture

For culture of *P*. *berghei* schizonts, cardiac blood at 2–3% blood stage parasitemia was collected from 2 to 4 Swiss Webster mice and was incubated in RPMI-1640 (Invitrogen) supplemented with 10% FCS and gentamycin for 16–23 h, gently shaking at 80 rpm in culture flasks that were flushed with 5% CO_2_, 5% O_2_, 90% N_2_ as described previously [72].

### Staining of parasitized reticulocytes

To quantify the number of parasites developing in reticulocytes versus normocytes, mice were inoculated i.v. with 10,000 BP1-CON and BP1-KO schizont stage parasites, obtained from *in vitro* culture as described above. Blood smears of infected mice were stained using a Brilliant cresyl blue and Giemsa double staining technique described previously [56]. Briefly, microscope slides were coated with 0.3% Brilliant Cresyl Blue (BCB) in 95% ethanol and dried overnight. These slides were incubated for 15 min at room temperature to allow absorbance of BCB, followed by methanol fixation and standard Giemsa staining [56]. Parasites in BCB-staining cells and cells not stained with BCB were counted.

### Cell cycle length determination

Growth assays for cell cycle determination were started with i.v. injection of 10^8^ synchronized schizonts, obtained from overnight culture of BP1-CON and BP1-KO blood stage parasites as above. Giemsa-stained blood smears were performed hourly for the next 30 h and scored as to the percent of total infected cells that were rings/early trophozoites versus mid/late trophozoites. Since the G1-S transition in blood stage *Plasmodium* parasites occurs as the parasite transitions from early and mid-stage trophozoite [57], these counts are indicative of the time it takes for the parasite to go through its cell cycle. At ~ 21 hours, schizonts begin to develop and their sequestration meant we could only follow parasite growth up to this time [76]. Though *P*. *berghei* is synchronous for up to 2 cycles [77], we found that the synchronicity of the second cycle was not as tight, making it difficult to obtain accurate cell cycle data after the first cycle.

## Supporting information

S1 Fig Targeting strategy to generate *berghepain-1* knockout parasites. **A**. To generate the targeting plasmid pBP1-KO, 1.6 kb of *berghepain-1* 5’ UTR and 1.3 kb of *berghepain-1* 3’ UTR were cloned from gDNA and inserted into pDEF-hDHFR-flirte plasmid [71], upstream and downstream of the human dihydrofolate reductase (hDHFR) cassette. For transfection, the plasmid was digested with EcoR1 to allow for double homologous recombination. **B.** To generate the targeting plasmid pBP1-CON, 1.84 kb of the *berghepain-1* 5’ UTR, 1.56 kb of the *berghepain-1* ORF and 1.33 kb of the *berghepain-1* 3’ UTR were cloned from gDNA and inserted into pDEF-hDHFR-flirte upstream and downstream of the human dihydrofolate reductase (hDHFR) cassette. For transfection, the plasmid was digested with EcoR1 to allow for double homologous recombination. **C.** Following transfection, diagnostic PCRs were performed on clonal BP1-CON (BCON) and BP1-KO (BKO) lines, confirming correct 5'- and 3'-integration of the construct, and absence of *berghepain-1* ORF in the BP1-KO clones and presence of the hDHFR selection marker cassette. Two BP1-KO clones (clones 1 and 2) were characterized and used for experiments. Location of primers used for PCR analysis and sizes of PCR products are shown. See S1 Table for all primer sequences.
(TIF)

S2 Fig The falcipain-1 inhibitor YA29 verifies berghepain-1 deletion. **A.** Structure of BMV109, a broad-spectrum cathepsin probe, which labels most cysteine proteases [78] **B.** BP1-CON and BP1-KO mixed blood stage parasite pellets were lysed and incubated in presence of the falcipain 1 inhibitor YA29 [23] prior to labelling with Cy5-BMV109. The berghepain-1 band between 17 and 24 kDa decreases in intensity in presence of YA29 in the BP1-CON lysate, but is not present in the BP1-KO lysate.
(TIF)

S3 Fig Normal growth of *berghepain-1* knockout liver stage parasites after intradermal inoculation of sporozoites. Infections were performed by intradermal injection of 10,000 BP1-CON or BP1-KO clone 2 sporozoites into Swiss Webster mice. RT-qPCR analysis of liver RNA 40 h post infection found no significant reduction of BP1-KO growth in the liver, as measured by parasite 18S rRNA copy number.
(TIF)

S4 Fig Normal merosome formation in *berghepain-1* knockout liver stage parasites BP1-KO clone 1. HepG2 cell monolayers were infected with BP1-CON and BP1-KO parasites, and after 48 h of *in vitro* culture, the number of EEFs per 50 fields of view, and after 65 h the number of merosomes per 20 fields of view, were counted. Results of one representative experiment, of a total of 2 experiments, performed using BP1-CON and BP1-KO clone 1 is shown.
(TIF)

S5 Fig Generation of transgenic lines expressing C-terminally tagged *berghepain-1*. **A.** Schematic representation of the tagging construct pL2018 targeting *berghepain-1* by single cross-over homologous recombination, and the locus before and after tagging in reporter line 1037cl1, which contains *ama1-gfp-luciferase* expression cassette at the *230p* locus. The tagging construct contains C-terminal triple *c-myc* (orange box) and the *tgdhfr/ts* drug selectable marker cassette (black box). Double lines indicate the enzyme site used for construct linearization. **B**. Southern blotting analysis of pulsed field gel-separated chromosomes confirm correct integration of the tagging construct. Chromosomes of the berghepain-1-myc parasite line 2338 were hybridized using a 3’UTR *pbdhfr* probe that recognizes the construct integrated into *berghepain-1* locus on chromosome 13, the endogenous *dhfr/ts* gene on chromosome 7, and the *ama1-gfp-luciferase* cassette at *230p* on chromosome 3. **C.** Western blotting analysis of mixed blood stages of the berghepain-1-myc line 2338 and the parental line 1037cl1 and probed with anti-c-myc antiserum showing expression of berghepain-1-myc (60 kDa), and an unspecific band at ~75 kDa, serving as a loading control for the parental line.
(TIF)

S6 Fig Expression of berghepain-1 in liver stage parasites. HepG2 cells infected with berghepain-1-myc parasites were fixed at the indicated timepoints, probed with anti-CSP (green) and anti-c-myc (magenta). Host and parasite DNA were stained with DAPI (blue). Scale bars: 10 μm.
(TIFF)

S7 Fig Immunofluorescene assays of liver stage control parental parasites probed with anti-c-myc. HepG2 cells infected with control parasites (1037cl1) were fixed at the indicated timepoints, stained for CSP (green) and anti-c-myc (magenta), confirming specificity of the anti-c-myc antiserum. Host and parasite DNA were stained with DAPI (blue). Scale bars: 10 μm.
(TIF)

S1 Table Primers used for genotype analysis. (TIF)
