# Supplementary figures and images for: Deletion of the rodent malaria ortholog for falcipain-1 highlights differences between hepatic and blood stage merozoites
Source: PLoS Pathog. 2017 Sep 18;13(9):e1006586. doi: 10.1371/journal.ppat.1006586 (PMC5602738; doi:10.1371/journal.ppat.1006586)

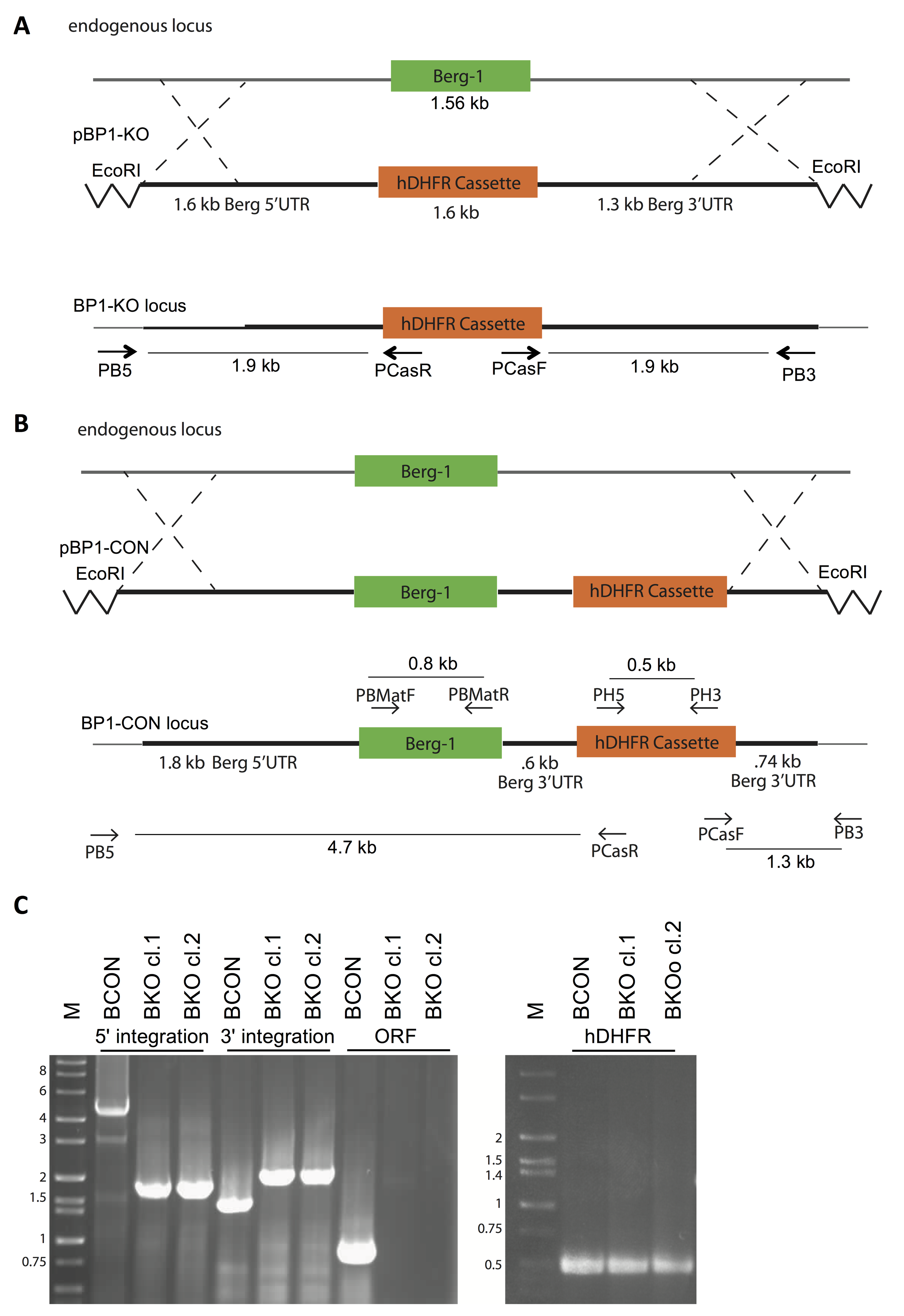

Supplement: S1 Fig — A. To generate the targeting plasmid pBP1-KO, 1.6 kb of berghepain-1 5’ UTR and 1.3 kb of berghepain-1 3’ UTR were cloned from gDNA and inserted into pDEF-hDHFR-flirte plasmid [71], upstream and downstream of the human dihydrofolate reductase (hDHFR) cassette. For transfection, the plasmid was digested with EcoR1 to allow for double homologous recombination. B. To generate the targeting plasmid pBP1-CON, 1.84 kb of the berghepain-1 5’ UTR, 1.56 kb of the berghepain-1 ORF and 1.33 kb of the berghepain-1 3’ UTR were cloned from gDNA and inserted into pDEF-hDHFR-flirte upstream and downstream of the human dihydrofolate reductase (hDHFR) cassette. For transfection, the plasmid was digested with EcoR1 to allow for double homologous recombination. C. Following transfection, diagnostic PCRs were performed on clonal BP1-CON (BCON) and BP1-KO (BKO) lines, confirming correct 5'- and 3'-integration of the construct, and absence of berghepain-1 ORF in the BP1-KO clones and presence of the hDHFR selection marker cassette. Two BP1-KO clones (clones 1 and 2) were characterized and used for experiments. Location of primers used for PCR analysis and sizes of PCR products are shown. See S1 Table for all primer sequences. (TIF) [file ppat.1006586.s001.tif]

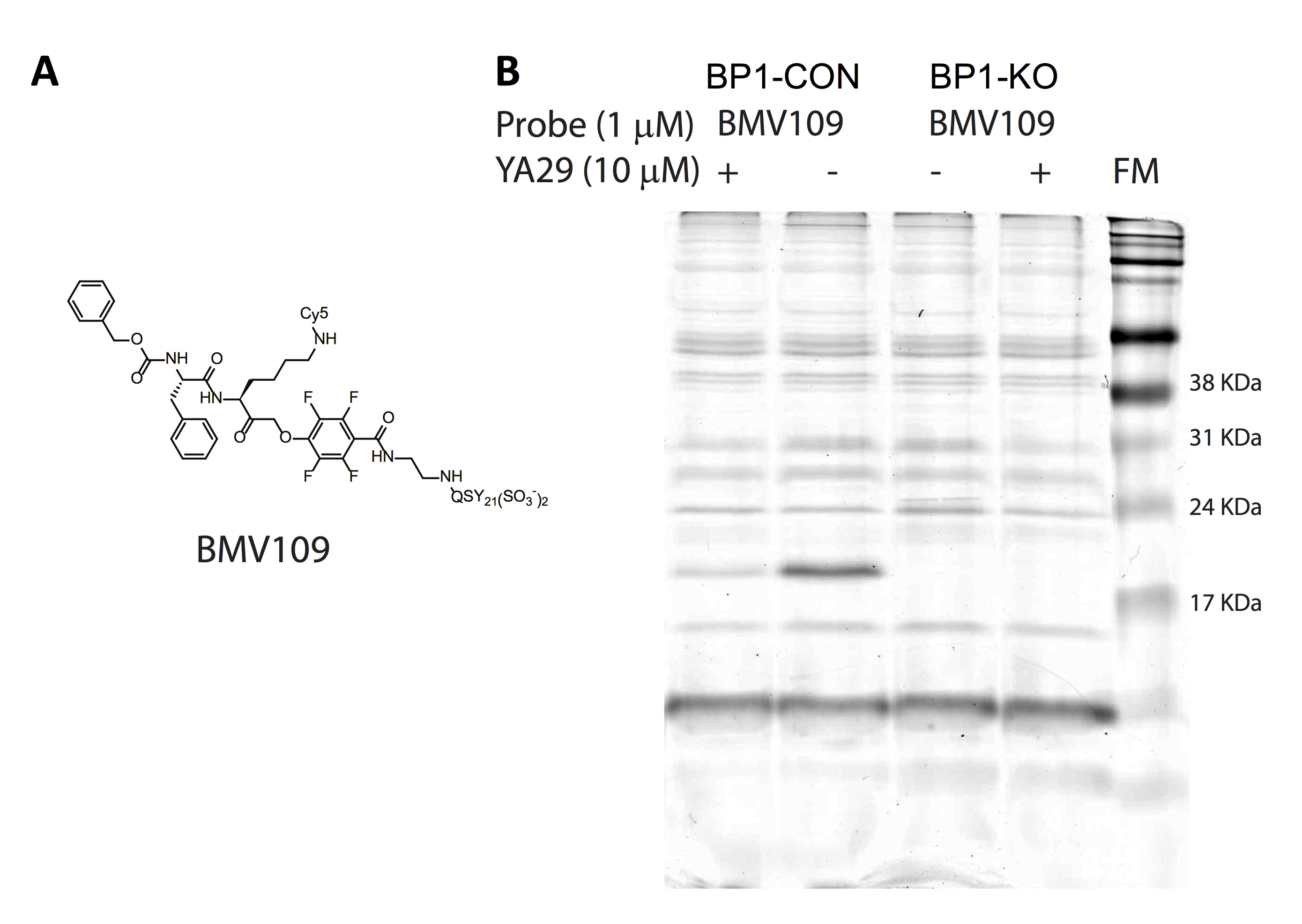

Supplement: S2 Fig — A. Structure of BMV109, a broad-spectrum cathepsin probe, which labels most cysteine proteases [78] B. BP1-CON and BP1-KO mixed blood stage parasite pellets were lysed and incubated in presence of the falcipain 1 inhibitor YA29 [23] prior to labelling with Cy5-BMV109. The berghepain-1 band between 17 and 24 kDa decreases in intensity in presence of YA29 in the BP1-CON lysate, but is not present in the BP1-KO lysate. (TIF) [file ppat.1006586.s002.tif]

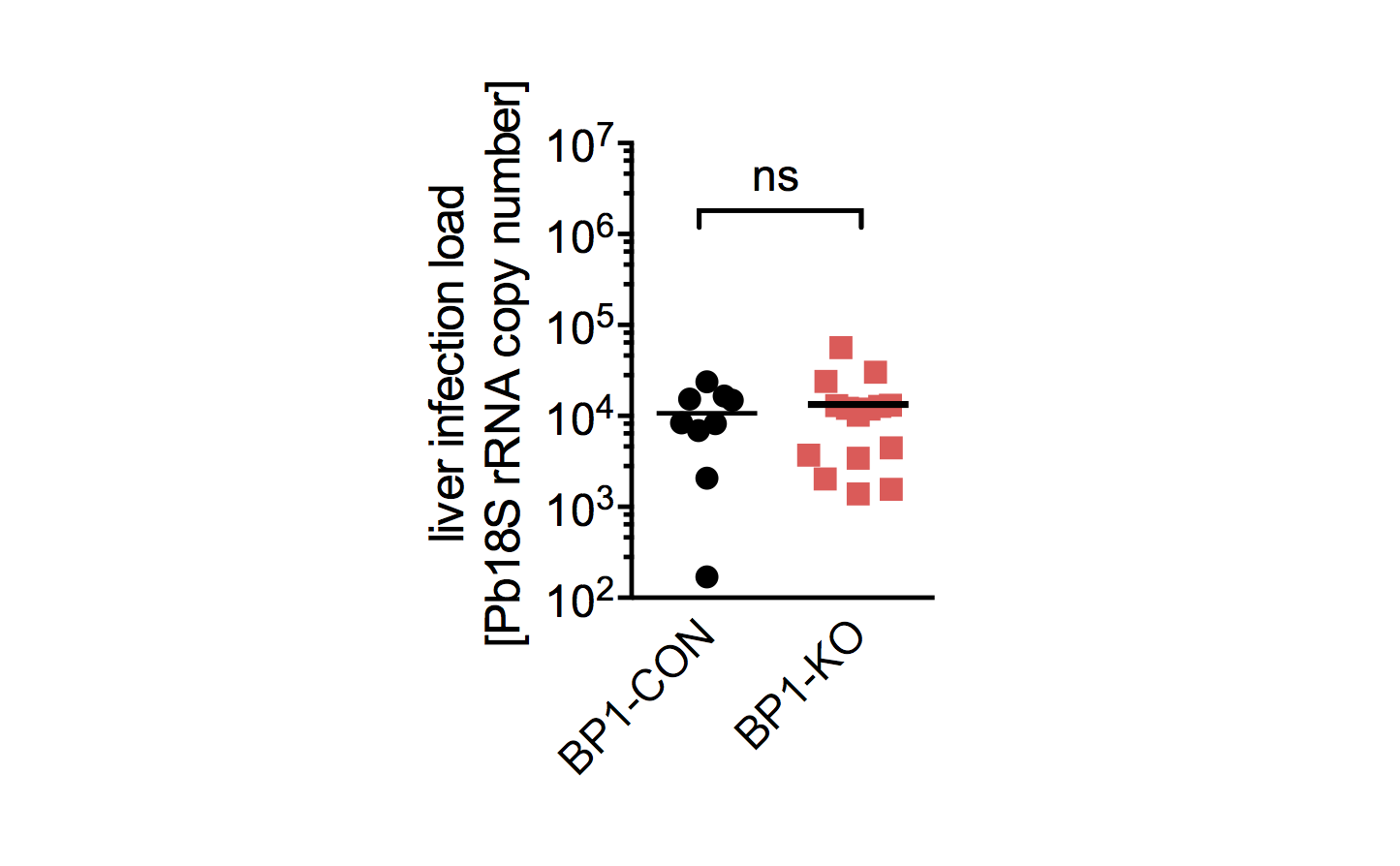

Supplement: S3 Fig — Infections were performed by intradermal injection of 10,000 BP1-CON or BP1-KO clone 2 sporozoites into Swiss Webster mice. RT-qPCR analysis of liver RNA 40 h post infection found no significant reduction of BP1-KO growth in the liver, as measured by parasite 18S rRNA copy number. (TIF) [file ppat.1006586.s003.tif]

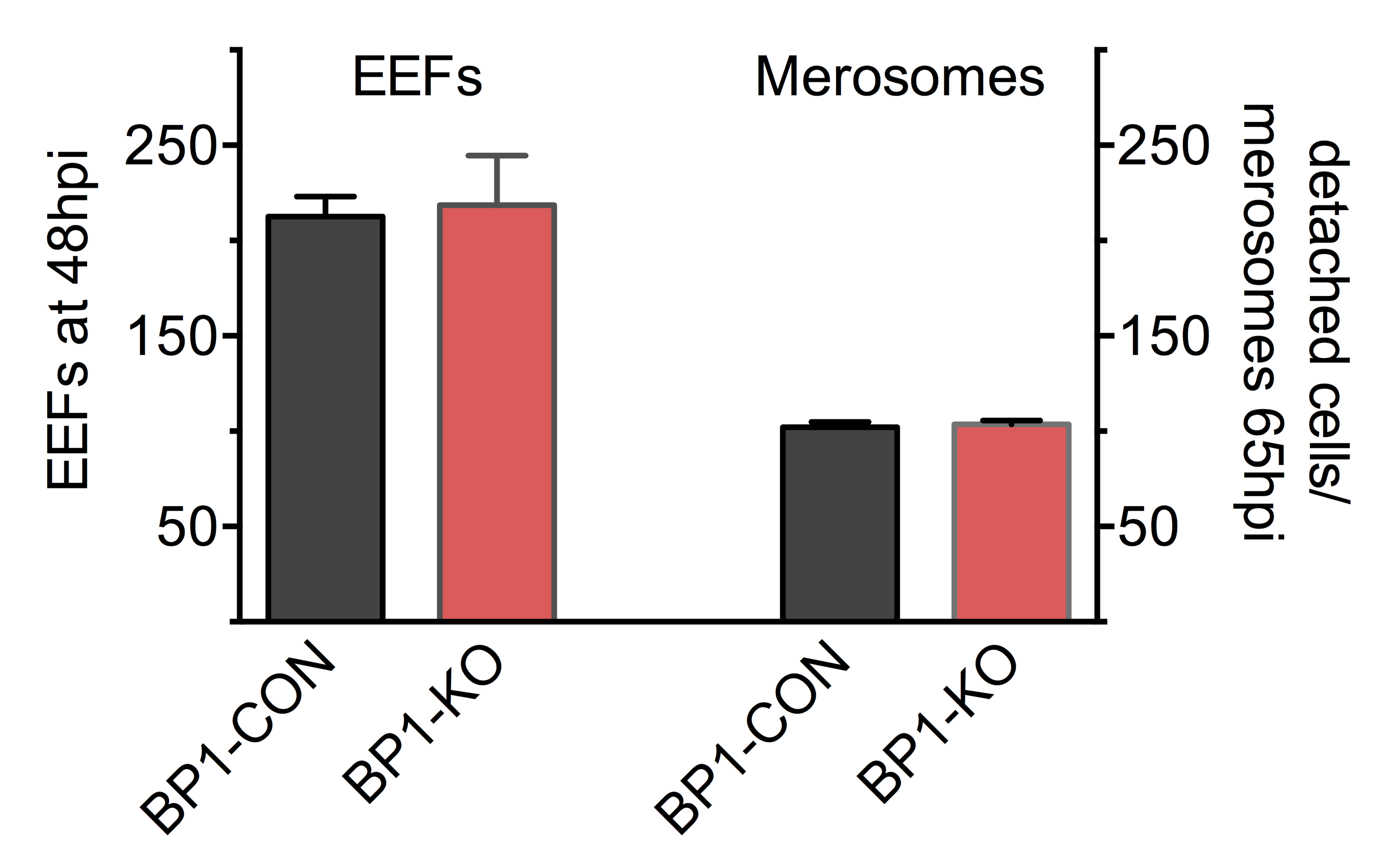

Supplement: S4 Fig — HepG2 cell monolayers were infected with BP1-CON and BP1-KO parasites, and after 48 h of in vitro culture, the number of EEFs per 50 fields of view, and after 65 h the number of merosomes per 20 fields of view, were counted. Results of one representative experiment, of a total of 2 experiments, performed using BP1-CON and BP1-KO clone 1 is shown. (TIF) [file ppat.1006586.s004.tif]

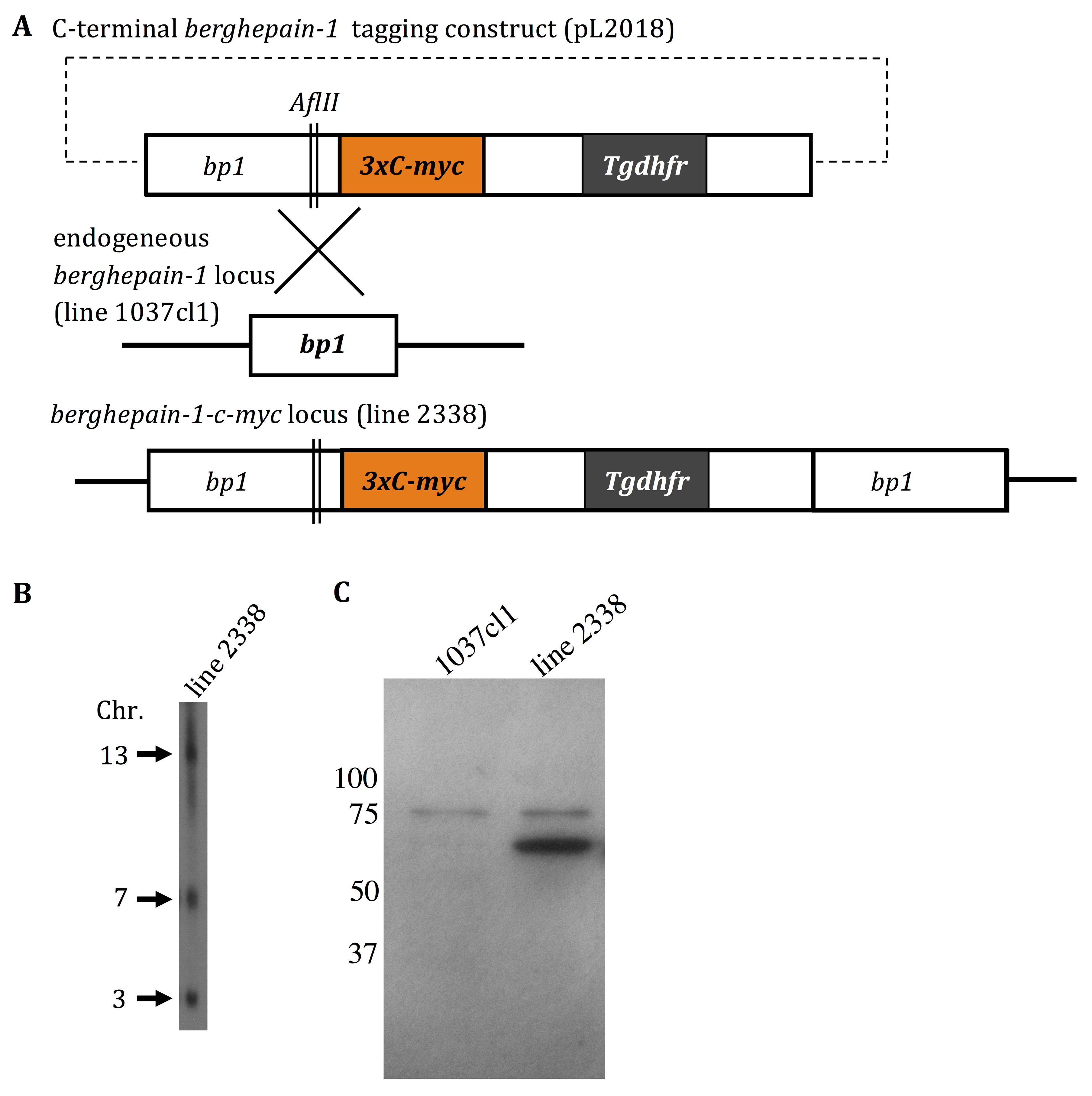

Supplement: S5 Fig — A. Schematic representation of the tagging construct pL2018 targeting berghepain-1 by single cross-over homologous recombination, and the locus before and after tagging in reporter line 1037cl1, which contains ama1-gfp-luciferase expression cassette at the 230p locus. The tagging construct contains C-terminal triple c-myc (orange box) and the tgdhfr/ts drug selectable marker cassette (black box). Double lines indicate the enzyme site used for construct linearization. B. Southern blotting analysis of pulsed field gel-separated chromosomes confirm correct integration of the tagging construct. Chromosomes of the berghepain-1-myc parasite line 2338 were hybridized using a 3’UTR pbdhfr probe that recognizes the construct integrated into berghepain-1 locus on chromosome 13, the endogenous dhfr/ts gene on chromosome 7, and the ama1-gfp-luciferase cassette at 230p on chromosome 3. C. Western blotting analysis of mixed blood stages of the berghepain-1-myc line 2338 and the parental line 1037cl1 and probed with anti-c-myc antiserum showing expression of berghepain-1-myc (60 kDa), and an unspecific band at ~75 kDa, serving as a loading control for the parental line. (TIF) [file ppat.1006586.s005.tif]

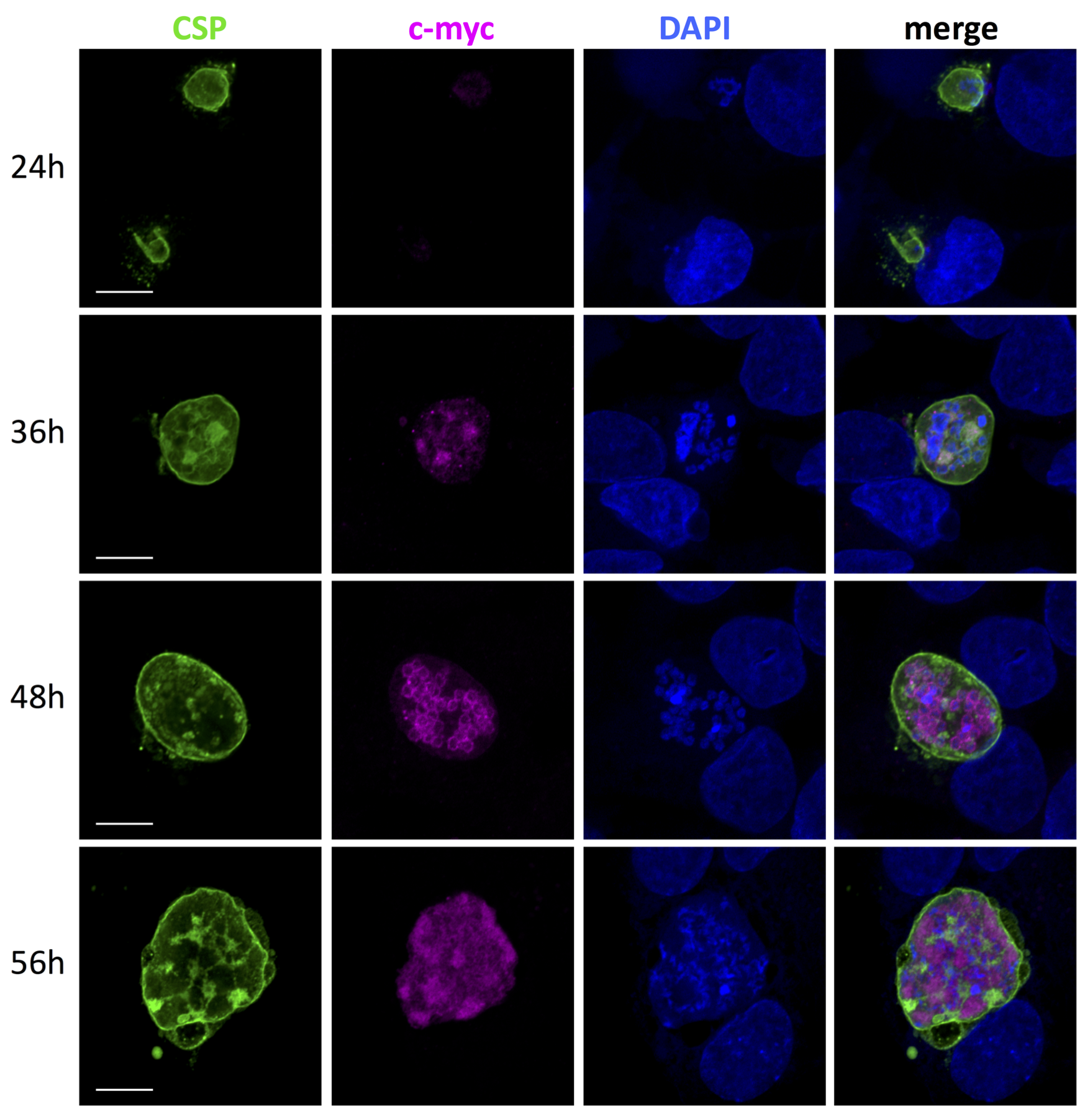

Supplement: S6 Fig — HepG2 cells infected with berghepain-1-myc parasites were fixed at the indicated timepoints, probed with anti-CSP (green) and anti-c-myc (magenta). Host and parasite DNA were stained with DAPI (blue). Scale bars: 10 μm. (TIFF) [file ppat.1006586.s006.tiff]

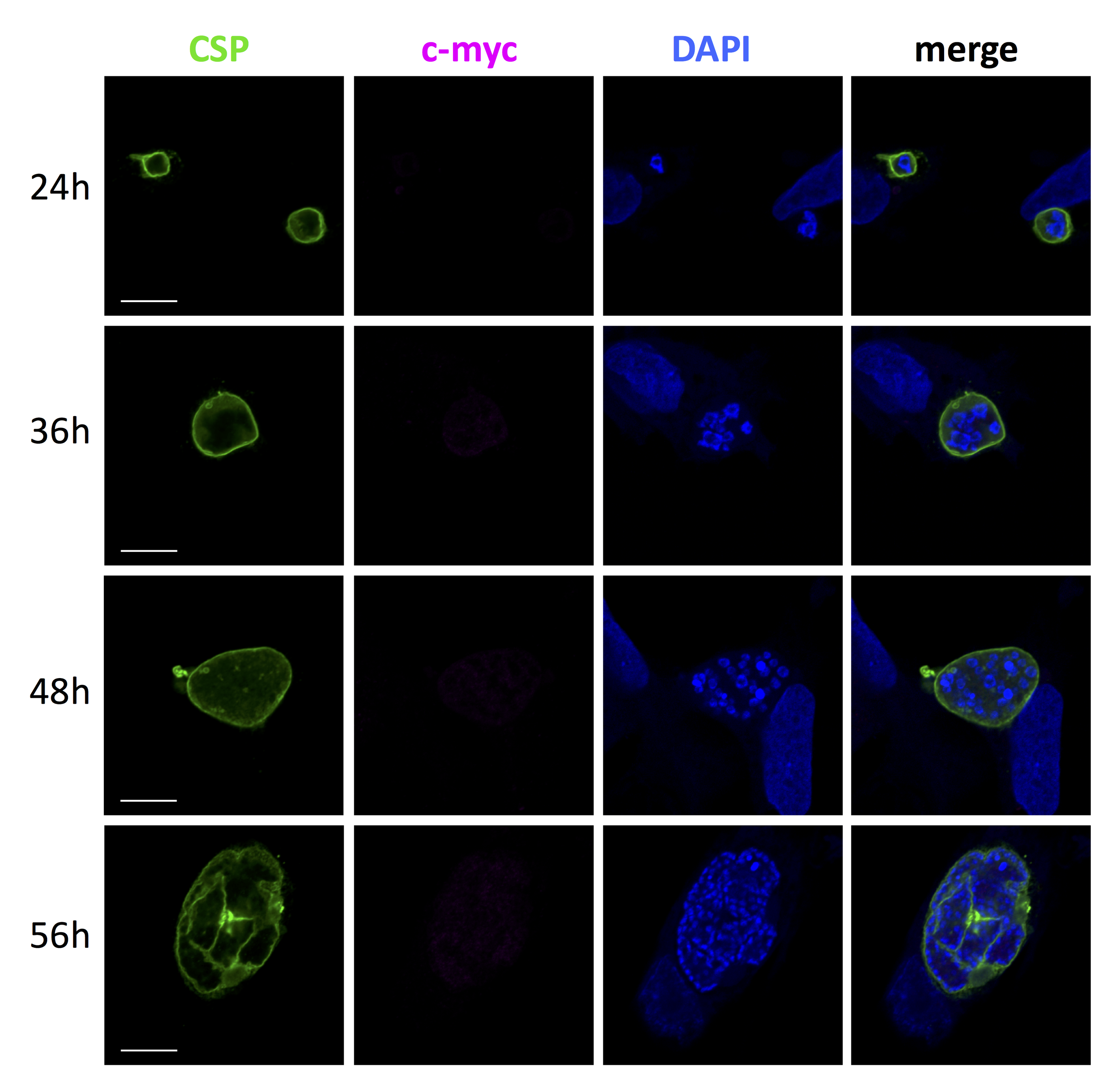

Supplement: S7 Fig — HepG2 cells infected with control parasites (1037cl1) were fixed at the indicated timepoints, stained for CSP (green) and anti-c-myc (magenta), confirming specificity of the anti-c-myc antiserum. Host and parasite DNA were stained with DAPI (blue). Scale bars: 10 μm. (TIF) [file ppat.1006586.s007.tif]

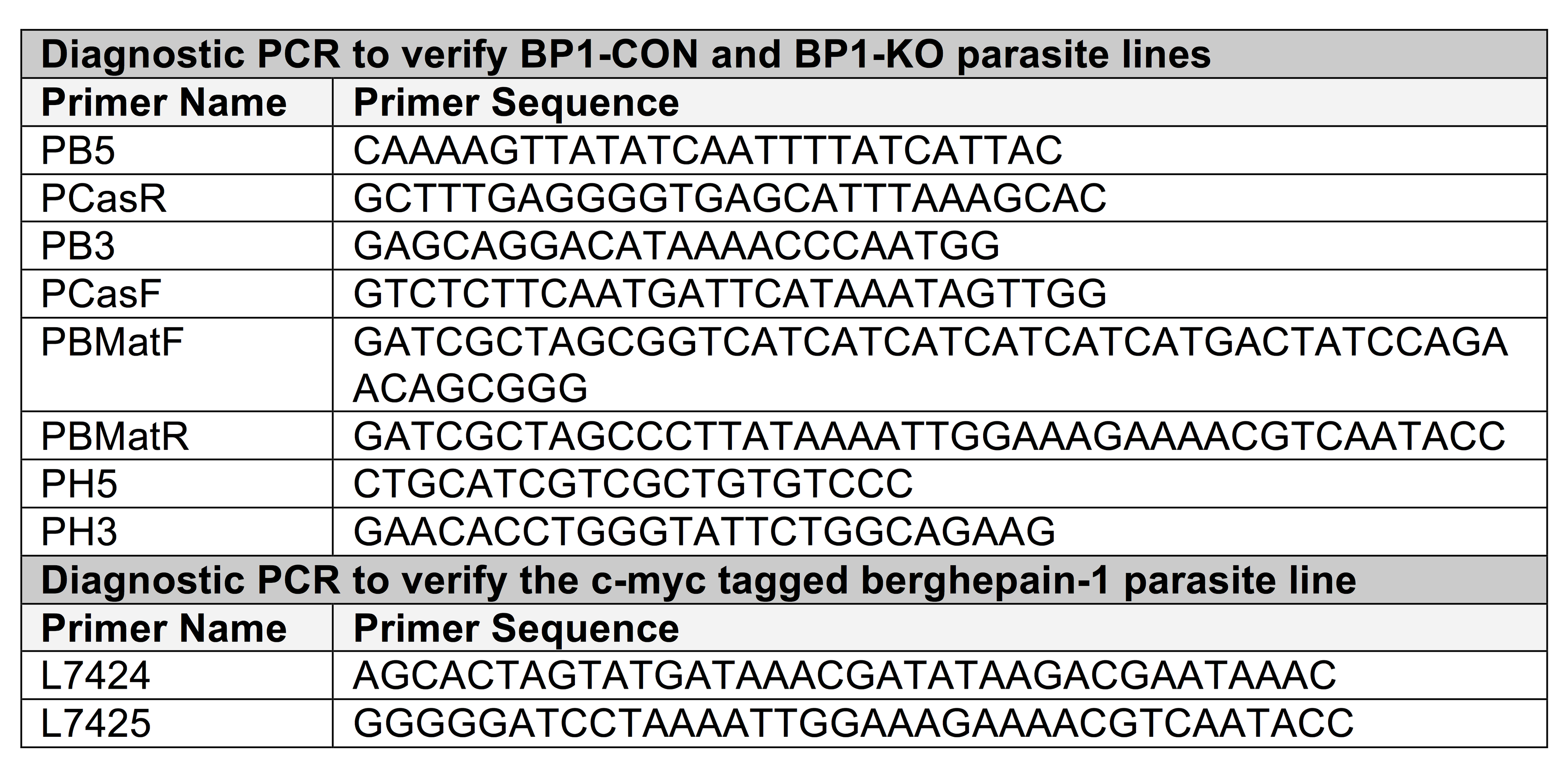

Supplement: S1 Table — (TIF) [file ppat.1006586.s008.tif]
